# An integrative characterization of recurrent molecular aberrations in glioblastoma genomes

**DOI:** 10.1093/nar/gkt656

**Published:** 2013-07-31

**Authors:** Nardnisa Sintupisut, Pei-Ling Liu, Chen-Hsiang Yeang

**Affiliations:** ^1^Institute of Statistical Science, Academia Sinica, Taipei, Taiwan, ROC and ^2^Institute of Information Science, Academia Sinica, Taipei, Taiwan, ROC

## Abstract

Glioblastoma multiforme (GBM) is the most common and malignant primary brain tumor in adults. Decades of investigations and the recent effort of the Cancer Genome Atlas (TCGA) project have mapped many molecular alterations in GBM cells. Alterations on DNAs may dysregulate gene expressions and drive malignancy of tumors. It is thus important to uncover causal and statistical dependency between ‘effector’ molecular aberrations and ‘target’ gene expressions in GBMs. A rich collection of prior studies attempted to combine copy number variation (CNV) and mRNA expression data. However, systematic methods to integrate multiple types of cancer genomic data—gene mutations, single nucleotide polymorphisms, CNVs, DNA methylations, mRNA and microRNA expressions and clinical information—are relatively scarce. We proposed an algorithm to build ‘association modules’ linking effector molecular aberrations and target gene expressions and applied the module-finding algorithm to the integrated TCGA GBM data sets. The inferred association modules were validated by six tests using external information and datasets of central nervous system tumors: (i) indication of prognostic effects among patients; (ii) coherence of target gene expressions; (iii) retention of effector–target associations in external data sets; (iv) recurrence of effector molecular aberrations in GBM; (v) functional enrichment of target genes; and (vi) co-citations between effectors and targets. Modules associated with well-known molecular aberrations of GBM—such as chromosome 7 amplifications, chromosome 10 deletions, EGFR and NF1 mutations—passed the majority of the validation tests. Furthermore, several modules associated with less well-reported molecular aberrations—such as chromosome 11 CNVs, CD40, PLXNB1 and GSTM1 methylations, and mir-21 expressions—were also validated by external information. In particular, modules constituting *trans*-acting effects with chromosome 11 CNVs and *cis*-acting effects with chromosome 10 CNVs manifested strong negative and positive associations with survival times in brain tumors. By aligning the information of association modules with the established GBM subclasses based on transcription or methylation levels, we found each subclass possessed multiple concurrent molecular aberrations. Furthermore, the joint molecular characteristics derived from 16 association modules had prognostic power not explained away by the strong biomarker of CpG island methylator phenotypes. Functional and survival analyses indicated that immune/inflammatory responses and epithelial-mesenchymal transitions were among the most important determining processes of prognosis. Finally, we demonstrated that certain molecular aberrations uniquely recurred in GBM but were relatively rare in non-GBM glioma cells. These results justify the utility of an integrative analysis on cancer genomes and provide testable characterizations of driver aberration events in GBM.

## INTRODUCTION

Glioblastoma multiforme (GBM) is the most common and malignant primary brain tumor in adults. Patients diagnosed with GBM typically have short survival times (∼1 year) and poor prognosis. Similar to other cancers, GBM cells harbor a large number of alterations at genetic, epigenetic, transcriptional and phenotypic levels [e.g. (1–7)]. The efforts of conducting a complete and comprehensive survey of cancer genomes were culminated in the Cancer Genome Atlas (TCGA) project (8).

The massive amount of omic data currently serve two primary purposes. First, molecular aberrations on DNAs, RNAs and proteins serve as biomarkers to categorize tumors into subclasses and predict prognosis, treatment efficacy and other clinical outcomes. GBMs are characterized by recurrent molecular aberrations including chromosome 7 amplification and chromosome 10 deletion [e.g. (9–11)], mutations of EGFR, PTEN, TP53, NF1 and IDH1 [e.g. (2,8)]. Panels of mRNA expression and DNA methylation profiles were used to predict clinical outcomes [e.g. (3–7)]. Beyond interrogation of individual genes, researchers also investigated alterations of pathway activities in brain tumors [e.g. (12–14)].

Second, it is of great interest to unravel causal and mechanistic relations regarding molecular aberrations in tumor cells. Among a large number of molecular alterations, only a small fraction of them may drive malignancy of cancers. The remaining alterations are likely passengers caused by chromatin instability and dysregulation of the transcriptional/translational apparatus. Separating driver from passenger aberrations and identifying the causal and mechanistic links connecting them are two key questions in cancer genomics. Many studies attempted to decipher the causal/regulatory relations of genes from expression data and external information [e.g. (15–20)].

Central dogma imposes a strong constraint on information flows from DNAs to proteins. Accordingly, many researchers seek associations connecting putative drivers on DNAs and passengers on mRNAs or proteins. Expression quantitative trait loci (eQTL) studies treat gene expression levels as traits and build association links with sequence variations on adjacent (*cis*-acting) or distant (*trans*-acting) loci [e.g. (21,22)]. In cancer genomes, there are rich studies building associations between copy number alterations (CNA) and mRNA expressions. Most of them identify *cis*-acting associations between amplified/deleted DNA segments and up/downregulated genes on the same or adjacent loci [e.g. (23–27)]. Some of these studies capture both *cis*-acting and *trans*-acting associations in the same modeling framework [e.g. (28–30)]. Beyond copy number variation (CNV) and mRNA data, there are several studies incorporating mutations, microRNA expression, DNA methylations and protein interaction networks in the models [e.g. (8,31,32)]. Despite the utility of these methods, they suffer from two shortcomings. First, although some of these approaches can in principle incorporate multiple types of high-throughput data in the same modeling framework [e.g. (28–30,32)], none of them explicitly unifies the data of mutation, CNV, single nucleotide polymorphism (SNP), DNA methylation, mRNA and microRNA expressions in the same model. Second, different types of associations need to be prioritized, as they carry disparate levels of mechanistic information. This issue is either irrelevant (e.g. when only CNV and mRNA data are considered) or not addressed in the prior studies.

Recently, we proposed a modeling framework to build ‘association modules’ from integrative cancer genomic data sets (33,34). The aim was to find statistical and causal links connecting molecular aberrations on DNAs or microRNAs (sequence mutations, CNVs, DNA methylations, SNPs, microRNA expressions) to the expressions of protein-coding genes. In this work, we apply this modeling framework to reconstruct the association modules from the integrated TCGA GBM data. Compared with our previous publications, the contribution of this work has two folds. First, it systematically invokes six validation tests with both reported knowledge and external data sets of central nervous system tumors to justify the relevance of these modules pertaining to the underlying regulatory mechanisms and prognosis of GBM. Second, the inferred association modules confirm prominent molecular aberrations of GBM and also report the influences of less well-known molecular aberrations and reveal their strong prognostic power. These results justify the utility of an integrative analysis on cancer genomes and provide testable characterizations of driver aberration events in GBM.

## MATERIALS AND METHODS

### Data sources and processing

The following multi-modal GBM data were downloaded from the TCGA data portal website (https://tcga-data.nci.nih.gov/tcga/): (i) an Affymetrix and an Agilent mRNA expression microarray data; (ii) two Agilent Comparative Genomic Hybridization (CGH) CNV array data; (iii) sequence data of 496 genes from three sources; (iv) one Illumina DNA methylation microarray data; (v) one Affymetrix and one Illumina SNP array data; (vi) one Agilent microRNA array data; and (vii) clinical information including ages, genders, dates of diagnosis and death (if applied), histological types, treatments of patients and others. Supplementary Table S1 summarizes the centers and platforms generating each type of data. Fifty-three genes were included in the mutation data, as they were probed in at least 50 samples and mutations in at least three samples were observed. Eight hundred seventy-five genes were included in DNA methylation data, as they were probed in at least 50 samples, and the numbers of hypo and hyper-methylated samples exceeded 10. The expression profiles of 22 697 mRNAs and 817 microRNAs were reported.

Each type of GBM data consists of distinct numbers of genes and samples. To generate a compatible joint data set for integrative analysis, we chose 248 samples appeared in all seven types of data and considered the union of all genes probed in each type of data. Supplementary Table S2 lists the TCGA IDs of the 248 selected samples and the NCBI symbols of 22 697 selected genes and 817 microRNAs.

Both mRNA and CNV data consist of two replicates generated by distinct laboratories. In both mRNA and CNV data, probes of the same genes between the replicates possess significantly higher correlation coefficients than those generated from randomly selected probe pairs (Supplementary Figures S1 and S2). Consequently, we merged multiple instantiations of mRNA expression or CNV data and generated a single data set for each type. For mRNA expressions, we rank-transformed the probe data of each data set into cumulative distribution function (CDF) values, averaged over the intra-gene CDF values on the same samples and then averaged the gene-level CDF values from the two data sets.

We formulated the associations between molecular aberrations and gene expressions with logistic regression models (see the text later in the text and Supplementary Text 1). To establish a unified format of data for the models, each type of data was transformed into probabilities of discrete states. For data types of mRNA and microRNA expressions, CNVs, DNA methylations, we proposed a probabilistic quantization approach to preserve information of continuous values (34). Rank-transformed CDF values were mapped into tristate probabilities [P(upregulation),P(nochange),P(downregulation)] by integrating over a family of monotonic quantization functions (see Supplementary Text 1). The data of mutations and SNPs were by nature discrete; hence, each entry was expressed as a binary vector with the entire probability mass on the designated state.

As amplification/deletion events on DNAs often stretch over multiple contiguous probes of CGH arrays, adequate units of CNV data are contiguous segments rather than individual probes. We partitioned normalized CNV probe data into segments using a naive Bayes model. The model hypothesizes that the CGH array probes on the same segment have consistent values, and thus their CNV states all depend on a common hidden variable. An algorithm was devised to recursively partition each chromosome into segments that maximized the likelihood function of the CNV probe data. The partitioned boundaries from individual samples of the two replicate data sets were merged to form consistent segments. The detailed procedures of the partitioning algorithm are described in (34) and Supplementary Text 1.

In all, 1353 candidate regulators were extracted from three sources: transcription factors from the TRANSFAC database (35), cancer-related genes according to the annotations from the Online Mendelian Inheritance in Man (OMIM) database (36), transcription factors and signaling proteins from the FanTom database (37).

To verify the reproducibility of association modules, we downloaded eight data sets of mRNA expression and clinical information of CNS cancers from the NCBI Gene Expression Omnibus database. Supplementary Table S3 summarizes the information of external data sets.

### Associations between molecular aberrations with gene expressions

**Figure 1.**
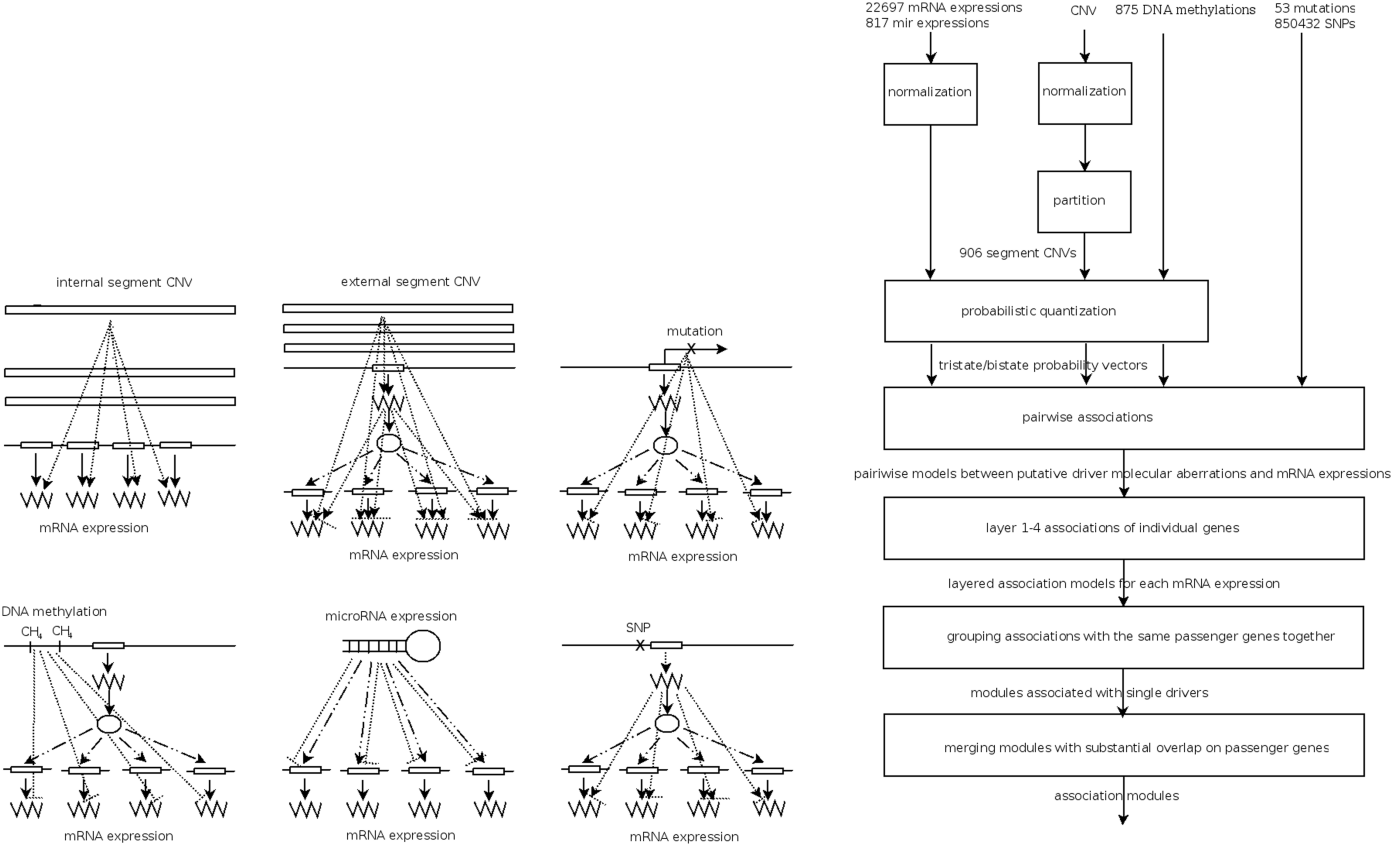
Left panel: Seven types of associations. From top-left to bottom-right: *cis*-acting effects with CNVs of internal chromosomal segments, *trans*-acting effects with CNVs of external chromosomal segments, associations with gene mutations, associations with DNA methylations, regulatory effects with microRNAs, *cis*- and *trans*-acting effects with SNPs. Solid lines: information flows from the central dogma (DNA → mRNA → protein). Dashed lines: regulatory links from regulators to their targets on other chromosomal locations. Dotted lines: associations between observed aberrations and mRNA/microRNA expressions. Arrowheads indicate positive associations and bar-ends indicate negative associations. External segment CNVs, mutations and SNPs can have both positive and negative associations. Internal segment CNVs only have positive associations. DNA methylations and microRNA expressions only have negative associations. Right panel: Flow chart of the algorithm for constructing association modules. Probe data of mRNA, microRNA expressions and CNVs are normalized to numbers within interval [0,1]. Normalized CNV data on each chromosome are partitioned into segments. The data with continuous values are converted into tristate probability vectors by probabilistic quantization. Large-scale pairwise associations between all putative effectors (segment CNVs, DNA methylations, microRNA expressions, mutations, SNPs) and target mRNA expressions are invoked. For each target, layered model selection is used to identify the effectors that explain its mRNA expression. Each layer (level) incorporates the effectors with a decreasing level of mechanistic information (see ‘Materials and Methods’ section for their definitions). Association modules are formed by grouping targets explained by each effector. Finally, modules sharing substantial fractions of targets are merged. The numbers of each type of effectors and targets are displayed.

We define an association module as a tuple consisting of three components: (i) observed effector molecular aberrations on DNAs or microRNAs; (ii) downstream target genes whose expression profiles are associated with effector molecular aberrations; and (iv) regulators (transcription factors or signaling proteins) that mediate the effects from effectors to targets. We consider the following seven types of associations and illustrate them in the left panel of Figure 1:

1. *Cis*-acting effects with CNVs of chromosomes*.* The CNV of a chromosome is positively associated with the expressions of its constituent genes.
2. *Trans*-acting effects with CNVs of chromosomes*.* The CNV of a chromosome manifests *cis*-acting effects with intermediate regulators, and both the chromosome CNV and regulator expressions are associated with the expressions of genes on other chromosomes. The direction of associations can be either positive or negative.
3. Effects with gene mutations*.* The mutational states of a gene are associated with the expressions of itself and other genes. The direction of associations can be either positive or negative.
4. Effects with DNA methylations*.* The coherent DNA methylation states of a collection of genes are negatively associated with the expressions of themselves and other genes.
5. Regulatory effects with microRNAs*.* The coherent expressions of a collection of microRNAs are negatively associated with the expressions of a collection of genes.
6. *Cis*-acting effects with SNPs*.* The SNPs on one or multiple adjacent loci are associated with the expressions of genes on the same chromosome.
7. *Trans*-acting effects with SNPs*.* The SNPs on one or multiple adjacent loci are associated with the expressions of genes on other chromosomes.

The expression profile of a target gene is possibly explained by one or multiple effectors. These associations are formulated as a logistic regression model. Denote **x** as effectors and y a target gene expression,
(1)
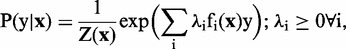

where 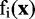 specifies the effect of the 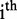 feature on y and 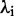 is a free parameter. Building an optimal association model is intractable due to combinatorial explosion. Therefore, we used two heuristics to streamline model selection. First, only the candidate effectors with significant marginal effects on the targets were considered. We incurred totally 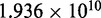 pairwise associations between candidate effectors and targets on 23 HP DL360 G7 servers in parallel. Each server contains dual Intel(R) Xeon(R) CPUs E5520 with 2.27 GHz and 24 GB main memory. The total running time was 50 h. In all, 2 135 755 pairwise associations were selected according to pre-determined thresholds of log-likelihood ratios, *P*-values and correlation coefficients reported in Supplementary Table S4.

Second, not all types of molecular aberrations are equally likely to drive gene expressions. Some candidate effectors provide direct explanations for gene expressions without requiring many mechanistic assumptions underlying gene regulation (e.g. *cis*-acting effects with CNVs). Others have massive number of features thus are likely to introduce spurious associations (e.g. SNPs). We proposed a layered modeling framework to prioritize molecular aberrations and incrementally incorporate candidate effectors to the model according to their priorities. Starting with an empty model without any effector, we incrementally selected the effectors that provided additional explanatory power relative to the preceding model. Candidate effectors were incorporated in the model with the following order of priority:

1. Level 1 associations: Mutations and DNA methylations of the target gene; segment CNVs covering or near (within 1 Mb) the target gene, SNPs on the same chromosome as the target gene.
2. Level 2 associations: Positive associations with segment CNVs on other chromosomes; positive and negative associations with non-local gene mutations; negative associations with non-local DNA methylations; associations with non-local SNPs.
3. Level 3 associations: Negative associations with segment CNVs on other chromosomes.
4. Level 4 associations: Negative associations with microRNA expressions.

**Figure 2.**
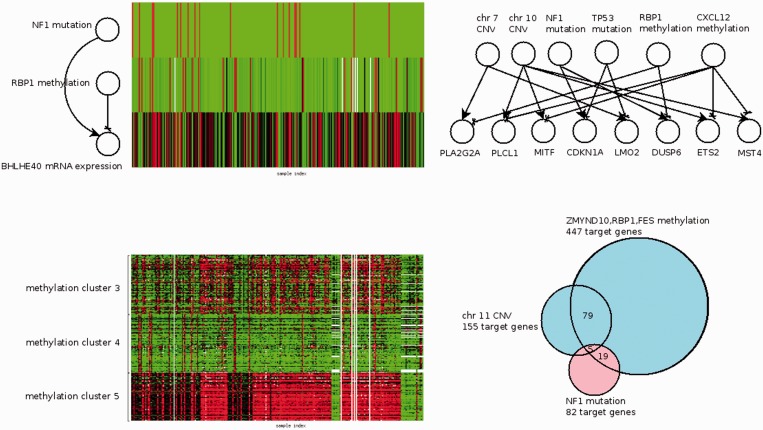
Illustration of association modules. Top-left panel: a simple association model with two effectors (NF1 mutation and RBP1 methylation) and one target (BHLHE40 mRNA expression). NF1 mutation and RBP1 methylation are positively and negatively associated with BHLHE40 mRNA expression. The measurement outcomes of those variables over 248 TCGA GBM samples are displayed as a heat map. Top-right panel: association models of six effectors and eight targets constitute a network. Bottom-left panel: DNA methylation profiles of 204 genes are grouped into three clusters. Bottom-right panel: Venn diagram of targets associated with three effectors: ZMYND10, RBP1 and FES methylation (447 target genes), chromosome 11 CNV (155 target genes) and NF1 mutation (82 target genes). The first two modules are merged as the size of their intersection (79 genes) exceed one-third of each module. The two merged modules are annotated with cyan and pink.

The top-left panel of Figure 2 demonstrates a simple joint association model. The mRNA expression of BHLHE40 is jointly associated with NF1 mutation (positive) and RBP1 DNA methylation (negative). The union of the association models for all target genes constitute a bipartite network with links between effectors and targets. A small association network is displayed in the top-right panel of Figure 2. In principle, each effector and its neighbor targets in the bipartite network comprise an association module. However, many modules will be highly overlapped due to similarity of effector profiles and shared targets. Consequently, we consolidated association modules with the following steps. First, closely related effectors were integrated: segment CNVs on the same chromosomes were combined, whereas DNA methylations and microRNA expressions were clustered by a graph theory-based clustering algorithm (see Supplementary Text 1). For instance, in the bottom-left panel of Figure 2, DNA methylation profiles of 204 genes form three clusters. Targets of the merged effectors were also merged. Second, in addition to resembled effectors, modules with considerable overlap of targets were also mergeable. Two modules were mergeable if their overlapped targets either exceed either one-third of the smaller module or 50 genes. A simplified example is shown in the bottom-right panel of Figure 2. The modules of chromosome 11 CNV and ZMYND10, RBP1 and FES methylation are mergeable as the intersected targets (79 genes) comprise more than half of the smaller module (155 genes).

The right panel of Figure 1 summarizes the procedures of association module construction. Detailed descriptions of data normalization, CNV segmentation, pairwise associations, layered model selection and assembly of association modules are reported in Supplementary Text 1. We also include the source codes of the module construction procedures in Supplementary Text 3.

### Validation of association modules

To justify the analysis results, it is essential to know whether the discovered association modules are statistically sound, biologically meaningful and reproducible in other data sets. We invoked six validation tests for each module to address these questions: (i) assessing the prognostic power of target gene expressions on survival times; (ii) verifying reproducible coherence of target gene expressions; (iii) verifying reproducible retention of effector-target associations; (iv) identifying recurrent effector aberrations in glioblastomas; (v) evaluating the functional enrichment of targets; and (vi) checking co-citations between effectors and targets.

### Assessing prognostic power of target gene expressions

In survival analysis, Cox regression coefficients gauge the dependency of patient’s hazard function on observed features (38). Positive associations with survival times exhibit negative Cox regression coefficients. Here, we treated the mRNA expression of each gene as a covariate and estimated its Cox regression coefficient in TCGA and three external data sets of brain tumors: GSE16011 (4), GSE4412 (5), GSE7696 (39). For each association module, the distribution of Cox regression coefficients in its targets was compared with the background distribution of all genes. The prognostic power of an association module was measured by the *P*-value of the one-sided Kolmogorov–Smirnov (KS) test between the two Cox regression coefficient distributions. The modules significantly enriched with predictive targets (*P*-value 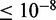) in at least three data sets were reported.

### Verifying coherence of target gene expressions

We evaluated the coherence of target gene expressions across the nine data sets. For each association module, we computed the distribution of correlation coefficients between target gene expressions in each data set. As a comparison, we extracted genes belonging to the Gene Ontology (GO) category of ribosomes (accession number 0005840) and computed the distribution of their expression correlation coefficients. The *P*-values of the one-sided KS tests between the distributions of target correlation coefficients and ribosome gene correlation coefficients were reported. Ribosome genes were chosen as a reference set, as they were strongly co-expressed across many tissue types and conditions (40). Consequently, the gene sets with higher correlation coefficients than the reference set should be strongly coherent.

### Verifying associations between effectors and targets

We examined the associations between effectors and targets across the nine data sets. Information about effector molecular aberrations (CNVs, mutations, DNA methylations) was not available in the external data sets. Therefore, we used the expressions of effector genes as proxies of their molecular aberrations. For chromosome CNVs, we chose the expressions of genes in the CNV *cis*-acting modules of the same chromosomes as proxies. For mutations and DNA methylations, we simply chose the expressions of the mutated/methylated genes as proxies. Associations with microRNA expressions were not considered as there were no mRNA proxies. For each module, the distribution of correlation coefficients between effector proxy and target expressions was evaluated. This distribution was compared with a background distribution of correlation coefficients between effector proxies and all valid genes. The *P*-values of the one-sided KS tests between the two distributions were reported.

### Identifying recurrent effector aberrations in GBM

We examined two expression data sets—GSE16011 (4) and GSE4412 (5)—containing multiple subtypes of gliomas including GBM and non-GBM samples. For each association module, we extracted the proxy expressions of their effectors and compared their distributions between GBM and non-GBM samples. The *P*-values of the two-sided KS tests between the two distributions were reported.

### Evaluating functional enrichment of target genes

We extracted 3312 functional categories from the GO database (41) and 889 pathways from three pathway databases: Reactome (42), BioCarta (43) and the NCI Pathway Interaction Database (44). We also extracted the 35 biomarker genes pertaining to prognosis of high-grade gliomas reported in (3) and divided them into three subclasses accordingly: proneural, mesenchymal and proliferative. In addition, we extracted 13 gene sets associated with embryonic stem (ES) cell identity from (45). For each association module, we applied the Fisher’s exact test to evaluate the enrichment significance of each GO category, pathway and gene set. The *P*-values were adjusted with Bonferroni correction by multiplying with the total number of GO categories, pathways or gene sets considered.

### Checking co-citations between effectors and targets

We incurred a batch search on the PubMed database to find all the pairs of effector/regulators and targets in each module that were co-cited in the same publications. For each effector/regulator in an association module, we counted both the numbers of co-cited genes among the target members and among all the 22 697 genes. Using the background frequency (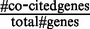) as a binomial probability of randomly finding a co-cited gene pair, we calculated the *P*-value of enrichment with co-cited target genes in a module.

Detailed procedures and results of each validation test are described in Supplementary Text 1.

## RESULTS

### A global landscape of molecular aberrations in glioblastoma

**Figure 3.**
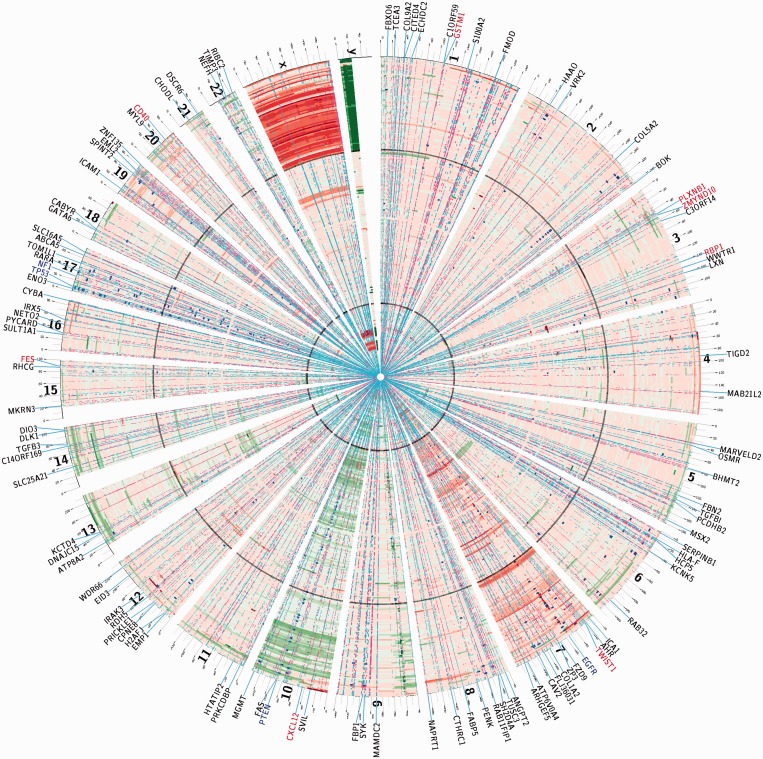
Circos plot of the global landscape of molecular aberrations in TCGA GBM. Each ring stands for molecular aberrations on the genome of one patient sorted by their chromosome coordinates. Patients are divided into three groups from the periphery to the center: 73 females, 120 males and 55 unannotated samples. Boundaries between groups are marked by black circles. Segment CNVs are color-coded with green (low CNV values) and red (high CNV values) stripes. Mutations of individual genes are marked by blue dots. DNA methylations are color-coded with cyan (hypo-methylation) and magenta (hyper-methylation) dots. Effectors and regulators of each association module are labeled on the periphery, with different colors representing distinct types of effectors or regulators: blue—mutated genes, red—methylated genes, black—regulators.

The circos plot (46) in Figure 3 displays three types of molecular aberrations—CNAs, gene mutations and DNA methylations—on the genomes of 248 GBM samples from the TCGA database (8). The CNV data—visualized as red (high values) and green (low values) stripes—of chromosomes X and Y are largely consistent with patients’ genders. Besides X and Y, chromosomes 7 and 10 undergo prevalent amplification and deletion, respectively. Other CNAs are more sporadic but tend to span the entire chromosomes rather than localize in small regions. Supplementary Figure S3 displays the auto-correlations of the CNV profiles between intra-chromosomal probe pairs segregated by their genomic distances. On most chromosomes, long-range correlations exist between distant probes. The results justify the use of one representative CNV profile for the CNV profiles of all probes on a chromosome.

Sequence mutations on protein-coding genes are categorized into two types: (i) nonsense point mutations or frame-shifting insertions/deletions that disrupt mRNA synthesis and (ii) missense point mutations or in-frame insertions/deletions that do not necessarily block mRNA synthesis. We term the first class as nonsense mutations, the second class as missense mutations and discard synonymous point mutations. Only five genes are mutated in at least 10 samples: IDH1, EGFR, PTEN, TP53 and NF1. The majority of NF1 mutations are nonsense (4 missense mutations and 14 nonsense mutations). In contrast, other genes are either dominated by missense mutations (IDH1, EGFR, TP53) or mixture of both types of mutations (PTEN).

The genotype data of 850 432 SNPs were reported. Owing to the large number of SNPs considered, we imposed a stringent threshold on filtering pairwise associations between genotypes and mRNA expressions (log likelihood ratio ≥7.0, *P*-value 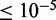). Only 15 *cis*-acting SNP–mRNA pairs pass the threshold. In all the 15 pairs, the SNP loci are within 50 kb from the mRNA genes. Owing to the small number of significant SNP–mRNA pairs, the *cis-* and *trans*-acting effects with SNPs are not incorporated in association modules but are visualized in Supplementary Figure S4.

### False-discovery rates of pairwise associations

The association modules were derived from pairwise associations between all putative effector molecular aberrations and target mRNA expressions. As a large number of hypotheses were tested, it is necessary to ensure that most significant pairwise associations do not arise by chance. We assessed the credibility of reported pairwise associations by false-discovery rates [FDR, (47,48)]. FDR estimates the fraction of false positives among the reported pairwise associations. We adopted the permutation tests described in (34) as the null model and evaluated two types of FDRs: (1) 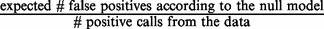 (48), 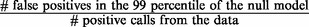 (2) (49). Table 1 shows the FDRs for each type of associations. Both types of FDRs on all pairwise associations combined are small (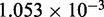 and 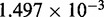, respectively). Associations with mutations yield the highest FDRs (0.171 and 0.230). The confidence of associations with mutations is likely degraded by the small numbers of samples carrying mutations. In contrast, the FDRs of all other types of associations are negligible, ranging from 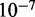 to 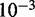.

**Table 1.** FDRs of pairwise associations

Type	FDR1	FDR2
*Cis*-acting CNV	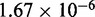	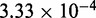
*Trans*-acting CNV	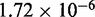	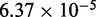
Mutation	0.17	0.23
Methylation	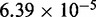	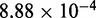
microRNA	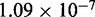	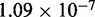
Total	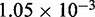	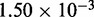

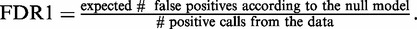

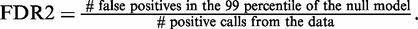

### Summary of association modules and their validation results

Table 2 and Supplementary Text 2 present the summary information of 45 association modules inferred from the integrated TCGA GBM data sets. In addition, we report the members of the association modules in an annotated webpage cancermodel.stat2.sinica.edu.tw/GBM/. Overall, 6331 genes belong to at least one association module. All types of molecular aberrations (except SNPs) appear as putative effectors to modulate mRNA expressions. Each chromosome constitutes an association module of *cis*-acting effects with CNVs (modules 5–28). Four modules contain multiple effectors (modules 1–4). Eight modules contain single CNV *trans*-acting effectors on chromosomes 7, 9, 11, 14, 19 and 20 (modules 29–36). Five modules contain single effectors of gene mutations (modules 37–41). Mutations of EGFR and TP53 exhibit both positive and negative associations with target mRNA expressions, whereas mutations of NF1 accommodate only positive associations. Two modules contain single effectors of DNA methylations: CD40 (module 42) and GSTM1 (module 43). Two modules contain effectors of microRNA expressions: one has mir-30c and mir-30e as effectors (module 44) and another has mir-215 as the effector (module 45).

**Table 2.** Summary information of association modules and their validation results

Index	Effectors	Sign	*N*	Function enrichment	Co-citation	CO	ET	RE	PR
1	chr10 trans CNV	+	767	Immune response	BLNK	6	8	chr10−	3+/1−
	TWIST1, CXCL12 methylation			Transmembrane receptor	CXCL12				
	mir-19a, mir-19b			Lysosomal membrane					
				Chemotaxis					
2	chr11 trans CNV	+	528	Inflammatory response	DDB2	8	8	RBP1−	4+
	ZMYND10, RBP1, FES methylation			Extracellular matrix	RBP1				
	mir-181a			Mesenchyme					
3	61 mirs	−	279	Olfactory receptor activity		6			
4	PLXNB1 methylation	−	806	Transcription regulation	PLXNB1	8	7		3−
	mir-21, mir-22			Nervous system development					
				H3K27 bound genes					
5	chr1 cis CNV	+	244		NA		NA		
6	chr2 cis CNV	+	14		NA		NA		
7	chr3 cis CNV	+	177		NA		NA		
8	chr4 cis CNV	+	63		NA		NA		
9	chr5 cis CNV	+	65	Protein binding	NA		NA		
10	chr6 cis CNV	+	134		NA	5	NA		
11	chr7 cis CNV	+	346	Mismatched DNA binding	NA	7	NA	chr7+	3+
				ATR pathway					
12	chr8 cis CNV	+	49	Ribonucleoprotein complex	NA		NA		
				Translational silencing					
				Translation initiation					
13	chr9 cis CNV	+	278		NA		NA		
14	chr10 cis CNV	+	263	Nanog, Oct4, Sox2 and NOS targets	NA	6	NA	chr10−	4−
15	chr11 cis CNV	+	149	Protein binding	NA		NA		
16	chr12 cis CNV	+	125	Protein binding	NA		NA		
17	chr13 cis CNV	+	135		NA		NA		
18	chr14 cis CNV	+	258	Cell cycle control	NA		NA	chr14−	
				Sox2, Myc targets					
19	chr15 cis CNV	+	160	Nanog targets	NA		NA		
20	chr16 cis CNV	+	105	Cell cycle control	NA		NA		
21	chr17 cis CNV	+	19		NA		NA		
22	chr18 cis CNV	+	35		NA		NA		
23	chr19 cis CNV	+	462	Transcription regulation	NA	5	NA	chr19+	
				Translational initiation, silencing					
24	chr20 cis CNV	+	184	Protein binding	NA		NA		
25	chr21 cis CNV	+	27		NA		NA		
26	chr22 cis CNV	+	185	Integral to membrane	NA		NA		
27	chrX cis CNV	+	12	Translation initiation	NA		NA		
28	chrY cis CNV	+	21		NA		NA		
29	chr7 trans CNV	+	117		EGFR		8	chr7+	3+
30	chr9 trans CNV	+	72		CDKN2A		7		
31	chr14 trans CNV	+	26		APEX1			chr14−	
32	chr19 trans CNV	+	122	Nervous system development			8	chr19+	
33	chr20 trans CNV	+	31	Regulation of signal transduction			8		
34	chr7 trans CNV	−	32				8		
35	chr10 trans CNV	−	249	Cell-cell adhesion	MGMT	5	9	chr10−	
				Cell cycle control					
				Nanog targets					
36	chr19 trans CNV	−	182	Nucleosome			9		
37	EGFR mutation	+	18	Phospholipid metabolic process					
38	EGFR mutation	−	15						
39	TP53 mutation	+	30	Myc targets					
40	TP53 mutation	−	48		TP53				
41	NF1 mutation	+	82	Keratinocyte differentiation	NF1	7	6		3+
				tnf/stress related signaling					
				Mesenchyme					
42	CD40 methylation	−	116	IL4-mediated signaling	CD40	8	8		3+
				Extracellular matrix					
43	GSTM1 methylation	−	39			5			3+
44	mir-30c, mir-30e	−	14				–	−	
45	mir-215	−	21				–	−	

The sign indicates the directions of associations between effectors and targets. N indicates the number of targets. Function enrichment: enriched GO categories/pathways/gene sets. Co-citation: effectors/regulators enriched with co-cited targets. CO: Coherence, number of data sets retaining target expression coherence. ET: Effector-target association, number of data sets retaining effector-target associations. RE: Recurrent effector, recurrent chromosome amplifications (+)/deletions (−) or DNA hyper-methylations (−)/hypo-methylations (+) in GBMs. PR: Prognosis, numbe of data sets enriched with positive (+) or negative (−) Cox regression coefficients on targets. The tests of co-citations and effector-target associations (ET) do not apply to CNV *cis*-acting modules (modules 5–28); thus the results are marked by NAs.

Table 2 also summarizes the validation results of association modules. Overall, 32 of 45 modules pass at least one validation test. Two modules pass all six validation tests: modules 1 and 2. Four modules pass five validation tests: modules 4 (PLXNB1 methylations and mir-181a expressions), 35 (chromosome 10 *trans*-acting CNVs), 41 (positive associations of NF1 mutations) and 42 (CD40 methylations). Three modules pass four validation tests: modules 11 (chromosome 7 *cis*-acting CNVs), 14 (chromosome 10 *cis*-acting CNVs) and 29 (chromosome 7 *trans*-acting CNVs).

Supplementary Table S5 reports the number and fraction of target genes and each type of effectors present in association modules. Each type of associations account for a relatively comparable number of target genes. In contrast, the number and fraction of each type of effectors exhibit great variations. Every chromosome constitutes a module of CNV *cis*-acting associations, and nearly one-third of all chromosomes (29.17%, seven chromosomes) constitute modules of CNV *trans*-acting associations. In contrast, only the mutations of three genes (5.66%) are associated with target gene expressions, and only the DNA methylation of eight genes (0.91%) are associated with target gene expressions.

The majority of prior studies integrating copy number and transcriptomic data considered only *cis*-acting effects of the same genes. Louhimo *et al.* (27) compared the performance of 10 algorithms capturing *cis*-acting CNV–mRNA associations. We applied six of those algorithms to the TCGA GBM data and compared the inferred genes putatively deregulated by CNVs with the genes manifesting *cis*-acting CNV–mRNA associations in our model. For each method, we sorted genes by their CNV–mRNA integration scores and counted the fraction of genes labeled with *cis*-acting CNV–mRNA associations in our model. Supplementary Figure S5 displays dependencies between the fraction of cumulative *cis*-acting gene numbers and gene ranks. The methods demonstrating the highest specificity in (27) are close to the *cis*-acting association modules [Pearson's correlation coefficient (PCC) and sparse partial least squares regression (sPLS)], whereas the methods demonstrating the highest sensitivity are dissimilar to the *cis*-acting association modules [statistical integration of microarrays (SIM) and similarity-constrained probabilistic canonical correlation analysis (pSIMCCA)]. The comparison results imply that the *cis*-acting association modules are likely to include most true positive associations as well as some false-positive associations.

### CNVs of chromosomes 7, 10, 11, mutations of NF1 and methylations of PLXNB1, CD40 and GSTM1 exhibit prognostic power in brain tumors

We extracted four expression data sets of CNS tumors and assessed the prognostic power of each gene expression profile by calculating its Cox regression coefficient and *P*-value (38) with respect to survival times. The prognostic power of an association module was evaluated by comparing the Cox regression coefficient distribution of its targets with the background distribution of all genes (see ‘Materials and Methods’ section and Supplementary Text 1 for detailed descriptions).

**Figure 4.**
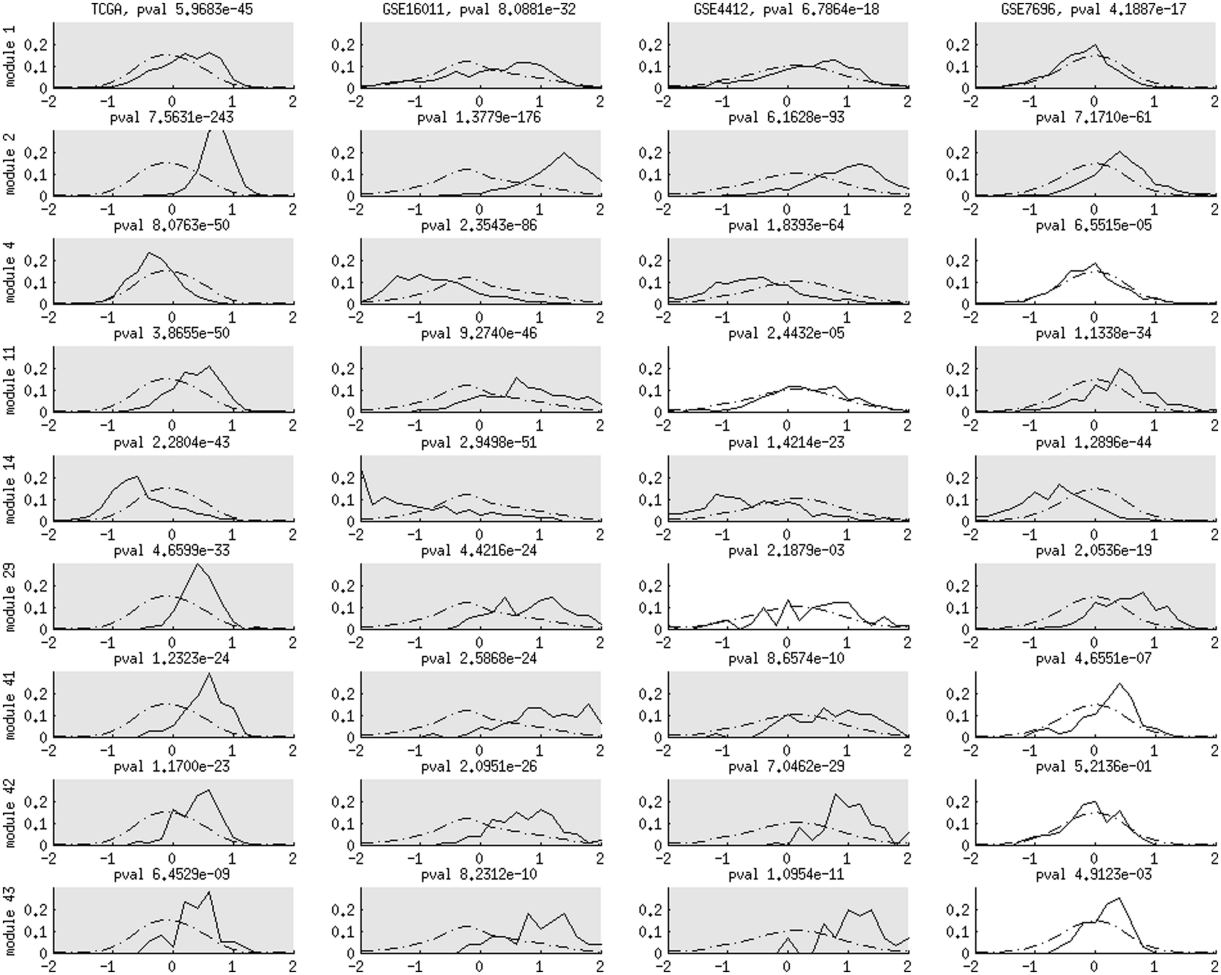
Cox regression coefficient distributions of nine association modules (rows) in four CNS data sets (columns). Solid curves display the Cox regression coefficient distributions of target genes in each association module. Dashed curves indicate the background distributions of all valid genes in each data set. The Cox regression coefficient of each gene expression profile contributes as one data point in the distributions. In each panel, the KS *P*-value of the deviation between the two distributions is reported. A panel is marked with grey if the corresponding Cox regression coefficient distribution significantly deviates from the background distribution (*P*-value 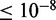).

Nine modules exhibit significant prognosis (*P*-value 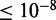) in at least three datasets (Figure 4 and Supplementary Table S6). Target expressions modulated by CNVs of chromosomes 11 (module 2), 7 (modules 11 and 29), NF1 mutations (module 41), methylations of CD40 (module 42) and GSTM1 (module 43) are negatively associated with survival times, as their Cox regression coefficients have positive deviations from the background distributions. Target expressions modulated by PLXNB1 methylations (module 4) and chromosome 10 *cis*-acting CNVs (module 14) are positively associated with survival times. Module 1 manifests negative Cox regression coefficients in GSE7696 but has a positive tendency in the remaining three data sets. The prognosis of these nine modules is independent of tumor types and grades in the Erasmus data (GSE16011) (Supplementary Figure S6).

**Figure 5.**
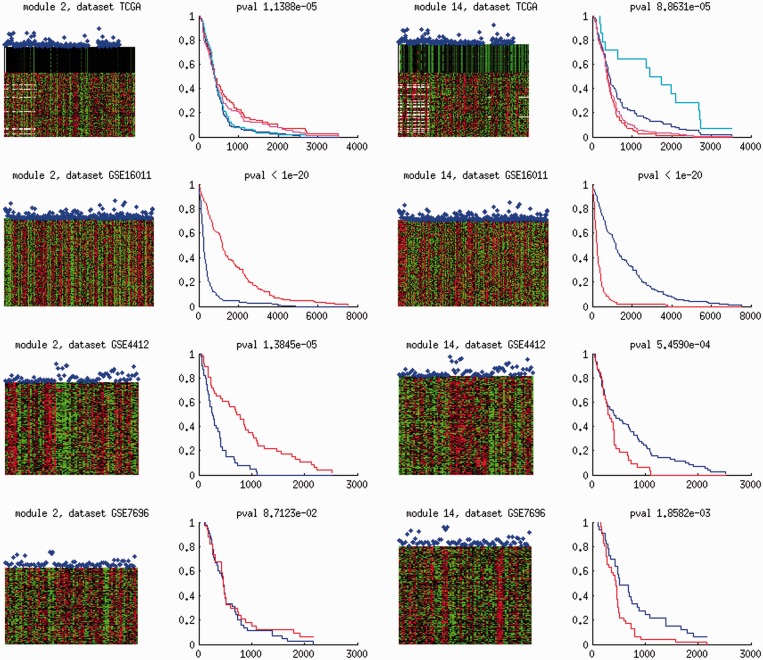
Molecular aberrations and survival information of modules 2 and 14 in four CNS data sets. Each row displays the information extracted from each data set. From left to right: (i) The heat map of module 2 target gene expressions in each module and survival times of patients. (ii) The Kaplan–Meier curves of patients stratified by the median of module 2 target expression values. (iii) The heat map of module 14 target gene expressions in each module and survival times of patients. (iv) The Kaplan–Meier curves of patients stratified by the median of module 14 target expression values. Horizontal and vertical axes in each heat map indicate the indices of samples (patients) and genes. Red and green colors indicate high and low expression values. For TCGA data, the levels of effector molecular aberrations in each module (chromosomes 11 and 10 CNVs, respectively) are displayed on top of target gene expressions. On top of each heat map, the relative survival durations of patients are shown as blue dots. In each module and each data set, a representative expression profile is generated by taking the median of the target expression levels. Patients are divided into groups with high (≥0.5) and low (<0.5) representative expression profile values. The Kaplan–Meier curves of the two patient groups are displayed: blue curves indicate the survival rates of patients with high expression levels, red curves indicate those of patients with low expression levels. Horizontal axis indicates days and vertical axis indicates the fraction of patients surviving beyond a fixed number of days. For TCGA data, the prognostic power of effector molecular aberrations is also displayed. Magenta curves indicate the survival rates of patients with high chromosome 11 or 10 CNV levels, and cyan curves indicate those of patients with low chromosome 11 or 10 CNV level.

Beyond the directions of associations with survival times, we also evaluated the strength of prognostic predictions for each module by the Cox regression coefficient *P*-values of the median expression profiles over their targets. Modules 2 and 14 manifest consistent and significant associations with survival times (*P* < 0.1) in all four data sets. Figure 5 visualizes the target expressions in modules 2 and 14 in relation to survival times and the Kaplan–Meier curves of patients segregated by the median target expression values. In TCGA data, the effector molecular aberration levels (chromosomes 11 and 10 CNVs) and their corresponding Kaplan–Meier curves are also displayed. Module 2 effector CNV and target expression levels have strong negative associations with survival times, whereas module 14 effector CNV and target expression levels have strong positive associations with survival times. The consistent and significant associations of modules 2 and 14 with survival times are also independent of tumor types and grades in the Erasmus data (Supplementary Figure S7).

Additional evidence strongly supports the prognostic relevance of modules 2 and 14. Module 2 harbors 7 of 11 genes involved in epithelial-mesenchymal transition (3). Patients with high expressions of these genes are previously reported to suffer from poor prognosis. We examined the Cox regression coefficients of these genes in each CNS data set and found they were all positive and largely significant except in GSE7696 (Supplementary Table S7). Furthermore, we identified 20 biomarker genes whose expression profiles yielded consistent (Cox regression coefficients have identical directions) and highly significant (Cox regression *P* ≤ 0.01) prognosis in all four data sets. In particular, module 2 consists of six biomarkers and module 14 consists of seven biomarkers.

### Association modules are aligned with GBM subtypes characterized by transcription and methylation profiles

Two prior studies provide comprehensive and robust molecular classifications of GBM samples. Verhaak *et al.* (6) used a panel of 840 gene expression profiles to divide GBM tumors into four classes: proneural, neural, classical and mesenchymal. Noushmehr *et al.* (7) found a distinct subset of GBM samples displaying concerted hyper-methylation at a large number of loci and named them as ‘glioma-CpG island methylator phenotype’ (G-CIMP). We compared the molecular characterizations derived from the association modules with the established GBM subtypes and found them considerably overlapped.

**Figure 6.**
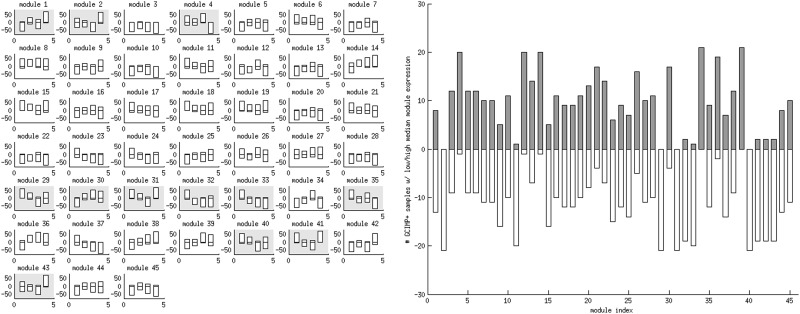
Left panel: Overlap counts of the four transcriptional subtypes and the binary classes obtained from the median target gene expressions in each association module. Four bars denote the overlap counts of classical (1st bars), neural (2nd bars), proneural (3rd bars) and mesenchymal (4th bars) subtypes. The bars on the negative/positive directions denote the numbers of samples with low/high target gene expressions in each association module. We define samples in a transcriptional subtype, and they are concentrated in one binary class if no more than 10 samples fall in its complementary binary class. Grey patches denote that at least one subtype is concentrated in each of the two binary classes. Right panel: Overlap counts of the G-CIMP positive phenotypes and the binary classes obtained from the median target gene expressions in each association module. Each bar denotes the overlap count for each module. White/grey bars in the negative/positive directions denote the numbers of G-CIMP positive samples with low/high target gene expressions.

For each association module, we built a binary classifier of GBM samples based on their median target gene expression levels. Overlaps of these classification outcomes and the four transcriptional subtypes are reported in the left panel of Figure 6 and Supplementary Table S8. Twelve association modules are remarkably aligned with at least two subtypes (yellow patches in Figure 6). For instance, in module 1, classical and mesenchymal subtypes have low and high target gene expressions, respectively (60 of 63 samples and 71 of 74 samples). The alignment outcomes are generally compatible with known genomic characteristics of the transcriptional subtypes. For instance, classical subtypes typically encounter chromosome 7 amplification (high expression of module 7 targets) and chromosome 10 loss (low expression of module 1 targets), whereas mesenchymal subtypes encounter NF1 mutation (high expression of module 41 targets). Supplementary Table S9 reports the dominant association module activities occurred in the samples of each transcriptional subtype.

We also counted the overlap of the G-CIMP subgroups (21 G-CIMP positive and 224 G-CIMP negative samples) with the binary classification defined by the median target gene expression of each association module. Intriguingly, although G-CIMP negative tumors are equally distributed between the two classes in all modules, G-CIMP positive tumors are biased toward high or low expressions (≥19 of 21 samples) in 16 of 45 modules. Nearly all the samples in the G-CIMP positive class possess low median expression levels in modules 2, 11, 29, 31, 32, 33, 40, 41, 42 and 43, and high median expression levels in modules 4, 12, 14, 34, 36 and 39 (the right panel of Figure 6, Supplementary Tables S9 and S10).

The superior prognosis of G-CIMP status explains away the strong associations of most modules with survival times. Conditioned on the G-CIMP status, the deviations of Cox regression coefficient distributions between the target gene expressions and background become insignificant in most modules (Supplementary Figure S8). This observation is compatible with prominent molecular characteristics of 16 association modules in G-CIMP positive tumors. The characteristic of each single module appears in most G-CIMP positive samples but many G-CIMP negative samples as well. However, concurrent presence of all 16 molecular characteristics is a much stronger indicator of the G-CIMP positive phenotype. Only 21 samples possess all the 16 molecular characteristics, and 14 are G-CIMP positive tumors (both precision and recall rates are 14/21 = 66.67%).

The presence of the joint hallmark is not perfectly aligned with G-CIMP positive status. Strikingly, unlike molecular characteristics of single modules, the prognostic power of the joint hallmark is not explained away by G-CIMP status. Supplementary Figure S9 displays the Kaplan–Meier curves of the four subclasses based on possible combinations of G-CIMP status and the joint hallmark. Although the presence/absence of the joint hallmark does not alter the already superior survival times of G-CIMP positive patients, G-CIMP negative patients possessing the joint hallmark have significantly longer survival times than those without the joint hallmark (median survival times are 633 and 372 days, respectively, logrank *P* ≤ 0.0356).

### Associations of effectors and targets are reproducible in external data sets

To validate reproducibility of association models, we incorporated eight additional gene expression data sets of CNS tumors (Supplementary Table S3). In each data set, we intended to check whether (i) targets in each association module retained coherent expression profiles and (ii) effector aberrations and target expression profiles remained associated with the directions compatible with the modules derived from the TCGA data.

Table 2 and Supplementary Table S11 report the target expression coherence of each association module in the nine data sets (including TCGA). Twelve modules retain coherent target expressions in at least five data sets (including TCGA). We were unable to directly verify associations between effector molecular aberrations and target gene expressions, as molecular aberration data were lacking in external data sets. Instead, we used the expression profiles of effectors as proxies for the molecular aberrations and examined their associations with target gene expressions. Table 2 and Supplementary Table S12 show the associations between the effector proxies and target gene expressions for each module across the nine data sets. Among the 21 association modules (excluding the 24 CNV *cis*-acting modules), 11 manifest significant effector-target associations (*P*-value 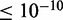) in at least six data sets.

### Amplification of chromosomes 7 and 19, deletion of chromosome 10 and RBP1 hypo-methylation are recurrent and specific molecular aberrations in glioblastoma tumors

Reproducible associations do not necessarily imply conserved molecular aberrations on effectors. It remains unclear whether molecular aberrations on certain effectors (i) recur on multiple GBM samples or (ii) are specific to GBM tumors. To answer these questions, we examined two expression data sets—GSE16011 (4) and GSE4412 (5)—including GBM and other glioma subtypes such as pilocytic astrocytomas, oligodendroglial tumors and mixed oligoastrocytic tumors. The goal is to identify the effector molecular aberrations that recur primarily on GBM but not on other glioma subtypes. As these molecular aberrations were not directly measured, we again used the effector expression profiles as their proxies. For chromosome CNVs, we used the expression profiles of the cis-acting targets as their proxies.

**Figure 7.**
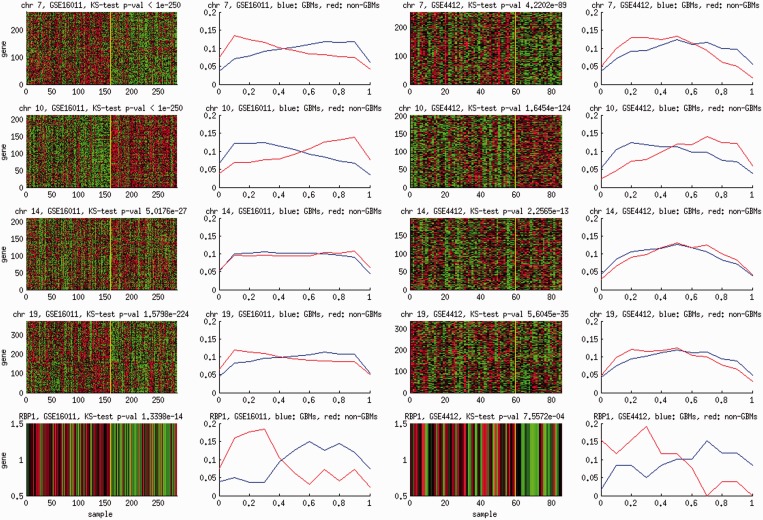
Molecular aberrations specific to GBM. The heat maps display the expressions of targets in the CNV cis-acting modules of chromosomes 7, 10, 14 and 19 and the expressions of RBP1 on two CNS data sets GSE16011 and GSE4412. GBM (left) and non-GBM glioma (right) samples are separated by yellow lines. The histograms of the expressions among GBM (blue curves) and non-GBM (red curves) samples are also displayed.

Figure 7 displays the GBM-specific recurrent effector molecular aberrations. Chromosomes 10 and 7 CNVs reveal the strongest contrasts between GBM and other glioma samples. In both data sets, the *cis*-acting targets of chromosome 10 have significantly lower expression levels in GBM than in other glioma samples (KS test *P*-values 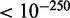 and 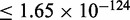, respectively). In contrast, the *cis*-acting targets of chromosome 7 have significantly higher expression levels in GBM samples (KS test *P*-values 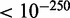 and 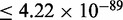, respectively). These results corroborate frequently reported chromosome 7 amplification and chromosome 10 deletion on GBM and furthermore indicate the uniqueness of these CNV aberrations on GBM.

Beyond chromosomes 7 and 10 CNVs, several other effectors also manifest GBM-specific aberrations. Chromosome 19 is amplified in GBM relative to other glioma samples. Chromosome 14 has moderately lower CNVs in GBM relative to other gliomas. RBP1 expression is upregulated in GBM and downregulated in other glioma samples, suggesting that it is hypo-methylated in GBM.

### Module targets are enriched with functional classes of immune/inflammatory responses, mesenchymal-epithelial transitions and ES cell transcription factor targets

We examined the enrichment of target genes of each association module on 3312 GO functional categories (41) and 889 pathways assembled from three sources (42,44). Table 2 and Supplementary Table S13 list the enriched functional categories/pathways and their hyper-geometric *P-*values after Bonferroni correction. In all, 25 of 45 association modules are enriched with at least one functional category/pathway. In particular, immune response genes are highly enriched in module 1 (*P*-value 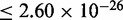), and inflammatory response genes are enriched in modules 1 and 2 (*P*-values 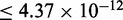 and 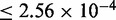, respectively).

Phillips *et al.* (3) used a 35-gene panel to cluster high-grade gliomas into three subclasses with distinct levels of prognosis: proneural, mesenchymal and proliferative. A disjoint set of genes are upregulated in each subclass. We found that modules 2 and 41 were highly enriched with genes expressed in the mesenchymal subclass (*P*-values 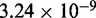 and 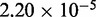, respectively). Notably, co-occurrence of NF1 mutations (the effector aberration of module 41) and upregulation of mesenchymal markers is previously reported (6).

Ben-Porath *et al.* (45) constructed 13 gene sets associated with embryonic stem (ES) cell identity and found that poorly differentiated tumors showed preferential overexpressions of the ES-associated genes. We found that module 14 was significantly enriched with the targets of key regulators of ES cell identity—*Nanog*, *Oct4*, *Sox2* and *NOS* (*P*-values 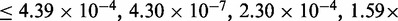 10^−6^ , respectively).

We also applied two widely used tools of functional enrichment—DAVID (50) and MSigDB (51)—to analyze the association modules and compared the results with our own hyper-geometric enrichment analysis (Supplementary Table S14). The results reported by DAVID are highly overlapped with Table 2 and Supplementary Table S13 as both are based on documented functional classes such as GO terms. For instance, the following functional classes are enriched according to both our analysis and DAVID: immune response in module 1, inflammatory response in module 2, olfactory receptor activity in module 3, mismatched DNA repair in module 11, protein complex assembly in module 15. In contrast, the enrichment results of MSigDB are ‘orthogonal’ to Supplementary Table S13, as the gene sets chosen (C2: curated gene sets and C6: oncogenic signatures) contain information not covered in GO terms and pathways. In particular, several gene sets related to brain function are enriched in multiple modules. Gene sets upregulated and downregulated in brain from patients with Alzheimer’s disease are enriched in 11 and 9 modules, respectively. Genes correlated with classical type of GBM are enriched in nine modules. Nevertheless, some gene sets are not obviously related to brain function but are still enriched in multiple modules, such as the differentially expressed genes in leukemia, fetal livers and breast cancers. Functional implications of these enrichment results need to be investigated.

### Co-citations between effectors and targets

We incurred a batch search for all pairs of effectors/regulators and targets of association modules on the NCBI PubMed database and checked whether they were co-cited in the same references. Table 2 and Supplementary Table S15 list the effectors with enriched co-cited target genes in each module. Overall, nine association modules contain enriched co-cited effector-target pairs.

### MicroRNA expressions are associated with CNVs of chromosomes 7, 10 and 11

Like protein-coding genes, microRNA expressions can also be modulated by effector molecular aberrations on DNAs. We used the module–discovery algorithm to microRNA expression data and identified 22 modules associated with *cis-*acting and *trans*-acting effects of CNVs (Supplementary Table S16). The size of each association module and the total number of microRNA expressions explained by CNVs are considerably smaller than those of mRNAs. The largest module consists of 19 target microRNAs, and only 114 microRNAs (13.95%) are targets in association modules, as compared with 6331 protein-coding genes (27.89%) included in association modules. However, the dominant chromosomes in CNV associations remain invariant between mRNA and microRNA modules: chromosomes 7, 10 and 11. The results suggest that the mechanisms of modulating RNA expressions through DNA copy number changes are likely similar between mRNAs and microRNAs.

## DISCUSSION

The inferred association modules simultaneously recapitulate critical molecular aberrations in GBM and map their presence and absence among predefined molecular subtypes. Table 3 combines the results of Table 2 and Figure 6 and summarizes the molecular aberrations in the four GBM transcriptional subtypes and G-CIMP positive tumors. GBM is typically characterized by chromosome 7 amplification, chromosome 10 deletion and NF1 loss-of-function mutations (9), (8,10,11). All these molecular aberrations become effectors of association modules. Beyond these well-known molecular aberrations, several other association modules reveal the effects of less well-known effectors: chromosome 11 deletion, chromosome 19 amplification, methylations of ZNYND10, RBP1, PLXNB1, CD40, GSTM1 and expressions of mir-181a, mir-21, mir-22. RBP1 is among the most differentially hyper-methylated and downregulated genes in G-CIMP positive tumors (7). PLXNB1 encodes plexin, a receptor for the semaphorin signals guiding axonal growth (52). In melanoma, PLXNB1 blocks tumorigenesis by inhibiting the MAP kinase pathway and controlling the extracellular matrix (53). CD40 encodes a TNF receptor essential in mediating a variety of immune and inflammatory responses (54). CD40 possesses multiple functions promoting tumorigenesis and progression including inflammatory response, repression of TNFα-induced apoptosis (55) and angiogenesis (56). GSTM1 encodes glutathione S-transferase mu 1, an enzyme catalyzing the biosynthesis of glutathione. Glutathione is an antioxidant protecting cells from the damage caused by reactive oxygen species. Previously, deletion and sequence polymorphisms on GSTM1 were reported in gliomas (57,58). Mir-21 is reported to have elevated levels in glioma cells (59) and can promote axonal growth as well (60).

**Table 3.** Molecular aberrations of the four GBM transcriptional subtypes and G-CIMP positive tumors

Classical	Neural	Proneural	Mesenchymal	G-CIMP +	G-CIMP + absent
chr 7 +	chr 7 +	chr 11 −	PLXNB1*^met^*	chr 11 −	chr 7 +
chr 9 −		chr 14 −	NF1*^mut^*	chr 14 −	chr 8 +
chr 10 −		ZMYND10*^met^*	mir-21/22 +	ZMYND10*^met^*	chr 10 −
chr 19 +		RBP1*^met^*		RBP1*^met^*	chr 19 +
TWIST1*^met^*		FES*^met^*		FES*^met^*	NF1*^mut^*
CXCL12*^met^*		CD40*^met^*		mir-181a +	PLXNB1*^met^*
CD40*^met^*		GSTM1*^met^*		CD40*^met^*	mir-21/22 +
		TP53*^mut^*		GSTM1*^met^*	
		mir-181a +		TP53*^mut^*	

The last column reports the molecular aberrations absent in G-CIMP positive tumors.

The target gene expressions of association modules are also aligned with documented characteristics of GBM molecular subtypes. For instance, in Table 3, classical subtypes possess pronounced chromosome 7 amplification and chromosome 10 deletion, proneural subtypes have TP53 mutations and mesenchymal subtypes harbor NF1 mutations (6). Moreover, alignment between association modules and CpG island methylator phenotypes reveals multiple concerted molecular aberrations on the G-CIMP positive samples. G-CIMP positive tumors typically lack chromosome 7 amplification, chromosome 10 deletion and NF1 mutation, but harbor methylations of RBP1, CD40 and GSTM1. Curiously, G-CIMP positive samples contain disproportionally frequent TP53 mutations (8 of 21 or 38.1%) compared with G-CIMP negative samples (35 of 224 or 15.62%).

Directions of associations between target gene expressions and survival times generally agree with the implicated consequences of effector molecular aberrations. Short survival times are likely caused by activation of oncogenes—EGFR amplification on chromosome 7 (4,5,61), mir-21 upregulation (59), CD40 hypo-methylation (55,56)—or inactivation of tumor suppressors—PTEN loss on chromosome 10 (2,8–11), NF1 mutation (2), (8), PLXNB1 hyper-methylation (53). In contrast, long survival times demand concurrent absence of all these molecular aberrations.

The functional consequences of molecular aberrations successfully explain the predictive power of the G-CIMP phenotypes for prognostic outcomes. Concerted hyper-methylation on CpG islands is a biomarker co-occurred with other benign molecular features (lack of chromosome 7 amplification, chromosome 10 loss, NF1 mutation, etc.). Therefore, the predictive power of each single module (Figure 4) is explained away by the G-CIMP phenotype (Supplementary Figure S8). Tumors carrying the joint hallmark of 16 modules are highly overlapped with G-CIMP positive tumors. In addition, G-CIMP negative tumors carrying the joint hallmark yield superior prognosis than the remaining G-CIMP negative tumors (Supplementary Figure S9). Furthermore, the joint expression hallmark derived from 16 association modules can be reduced to four modules without sacrificing its prognostic power (Supplementary Figure S10). Downregulation of modules 11 and 42 and upregulation of modules 12 and 14 suffice to segregate the 10 G-CIMP negative tumors with long survival times from the remaining G-CIMP negative samples. From these observations, we propose that a joint hallmark derived from 16 (or 4) association modules can faithfully predict GBM prognostic outcomes.

Another striking finding from our analysis is pronounced activities of the immune system in inferred modules and their significant associations with survival times. GBM is typically characterized with a strong immunosuppressive microenvironment (1,62,63). Chromosomes 10 and 11 CNVs are positively associated with expressions of many immunity or inflammation related genes on other chromosomes. Furthermore, effectors of modules 42 (CD40) and 43 (GSTM1) are also involved in inflammatory responses. Conventional wisdom often links chromosome 10 loss with inactivation of PTEN (2,8–11) and views activation of the PI3K/Akt/mTOR pathway through PTEN mutations as the primary cause of immune suppression in GBM (64,65). Our study suggests that PTEN might not be the only key regulator gene on chromosome 10, as neither PTEN expression nor PTEN mutation is strongly associated with target gene expressions. Other candidate regulators on chromosome 10 such as CXCL12 and BLNK retain strong associations with chromosome 10 CNVs and target gene expressions across multiple data sets. Alterations on those genes may directly modulate immune responses in addition to the effect of the PI3K/Akt/mTOR pathway.

Observations from survival analysis, however, are paradoxical. All the association modules enriched with immune/inflammatory response genes—modules 1, 2, 41 and 42—are also negatively associated with survival times. This observation seems to contradict with the nature of an immuno-deficient microenvironment of GBM. However, immune/inflammatory responses can both promote tumorigenesis by fostering angiogenesis, cancer cell proliferation and invasiveness (66) and suppress/attack cancer cells presenting specific antigens (63). In GBM, the cancer-promoting characteristics of immune/inflammatory responses seem to dominate the progression of tumors. These observations can be attributed to the canonical theory of the effects of inflammation on tumorigenesis, and recent findings about strong couplings of the KRAS signaling pathway and innate immune signaling (67). More refined investigation is required to determine the effects of antagonistic interactions for immune/inflammatory responses.

There are several limitations in the data and methods of the current study. First, intratumoral heterogeneity among cancer, stromal and immune cells is not considered. Interactions between multiple cell types are critical for tumor progression but cannot be unraveled with the population-averaged TCGA data. Second, prognostic prediction among the majority of G-CIMP negative patients remains poor. The wide range of survival times among this group (though generally lower than G-CIMP positive patients) cannot be further stratified by association modules or transcriptional subtypes. Third, causal and mechanistic links between molecular aberrations remain unknown, as longitudinal data of tumor evolution and intervention experimental data are unavailable.

To sum up, cross-sectional, static, observational and coarse-grained TCGA data supply rich information of molecular characteristics of cancer genomes. Yet, they are unable to tackle several central issues of contemporary cancer research, such as evolution of cancer genomes, heterogeneous interactions between tumors and microenvironment, emergence and development of cancer stem cells and acquisition of drug resistance. New experimental technologies and computational methods will enable scientists to study these questions and hopefully acquire systematic understanding and treatment strategies of cancer.

## SUPPLEMENTARY DATA

Supplementary Data are available at NAR Online, including (68).

## FUNDING

Academia Sinica (to N.S., P.L.L. and C.H.Y.); National Science Council in Taiwan [102-2221-E-001-030- and 100-2118-M-001-008-MY2 to C.H.Y.]. Funding for open access charge: National Science Council, Taiwan [102-2221-E-001-030-].

*Conflict of interest statement*. None declared.

## Supplementary Material

Supplementary Data
