# Supplementary material for: An integrative characterization of recurrent molecular aberrations in glioblastoma genomes
Source: Nucleic Acids Res. 2013 Jul 31;41(19):8803–21. doi: 10.1093/nar/gkt656 (PMC3799430; doi:10.1093/nar/gkt656)
Supplement: Supplementary Data [file supp_41_19_8803__index.html]

An integrative characterization of recurrent molecular aberrations in glioblastoma genomes — An integrative characterization of recurrent molecular aberrations in glioblastoma genomes — Supplementary Data 

# An integrative characterization of recurrent molecular aberrations in glioblastoma genomes

## 

files

**Files in this Data Supplement:**

- Supplementary Data - zip file
