# Supplementary material for: An integrative characterization of recurrent molecular aberrations in glioblastoma genomes
Source: Nucleic Acids Res. 2013 Jul 31;41(19):8803–21. doi: 10.1093/nar/gkt656 (PMC3799430; doi:10.1093/nar/gkt656)
Supplement: Supplementary Data [file supp_gkt656_nar-00272-n-2013-File002.zip › Yeang_GBM_NAR_suppl_rerevision.pdf]

An integrative characterization of recurrent molecular aberrations in glioblastoma genomes

Nardnisa Sintupisut[1], Pei-Ling Liu[2], Chen-Hsiang Yeang[1*]

[1]Institute of Statistical Science, Academia Sinica, Taipei, Taiwan, ROC.

[2]Institute of Information Science, Academia Sinica, Taipei, Taiwan, ROC.

[*]Corresponding Author: chyeang@stat.sinica.edu.tw.

Data sources and pre-processing

Data sources

Seven types of GBM data were downloaded from the TCGA data portal website (https://tcga-data.nci.nih.gov/tcga/) and included in analysis. The following types of data are included in analysis: (1)two mRNA expression datasets generated by Affymetrix and Agilent microarrays, (2)two CNV datasets generated by Agilent CGH arrays, (3)sequencing data of 53 genes from 3 sources, (4)one DNA methylation dataset generated by Illumina microarrays, (5)two SNP datasets generated by Affymetrix and Illumina SNP chips, (6)one microRNA dataset generated by Affymetrix trips, (7)clinical information including the ages, genders, dates of diagnosis and death (if applied), histological types, treatments of patients. Supplementary Table S1 summarizes the centers and platforms of each type of data.

Each type of data covers a highly overlapped but non-identical set of samples. We chose the intersection of the samples covered by all types of data. Overall there are 248 samples according to the data downloaded in May 2010. The symbols of 22697 genes, 817 microRNAs and IDs of the 248 selected samples are reported in the Supplementary Table S2.

TCGA provides four levels of data with an increasing extent of processing and interpretation. We used level 2 data in our analysis for they contained probe-level information of microarray data.

Both mRNA expressions and CNVs of the same set of samples were probed by two independent centers. The quality of replicated data was verified by evaluating the correlation coefficients between the probes of the same genes. Supplementary Figure S1 displays the intra-gene probe correlation coefficient distribution between the two mRNA expression datasets (generated Broad Institute and University of North Carolina). As a comparison we also randomly drew 500 probe pairs between the two datasets and evaluated the distribution of their correlation coefficients. The intra-gene correlation coefficients are considerably higher than the background distribution (the mean correlation coefficient 0.7), substantiating the consistency between the two datasets.

Similar to mRNA data, we evaluated the distribution of intra-gene probe correlation coefficients between the two CNV datasets (Harvard Medical School and Memorial Sloan-Kettering Cancer Center). Supplementary Figure S2 displays the intra-gene probe correlation coefficient distribution between the two CNV datasets and the background distribution. The overall intra-gene probe correlation coefficients are still substantially higher than the background.

The SNP data downloaded from TCGA were continuous values generated by microarrays. We applied Genotyping Console(TM) version 4.0 to convert the Affymetrix SNP array data into genotypes. The genotype data of the Illumina SNP array are directly provided by TCGA.

Data normalization

The TCGA GBM dataset consists of 6 types of data: gene mutations, copy number variations, DNA methylations, single nucleotide polymorphisms, microRNA expressions and mRNA expressions, and most data have multiple replicates generated from distinct member institutions of the consortium. To incorporate the heterogeneous sources of data in the same integrated model, we had to convert them into the same format with compatible scales. We treated

each feature as a discrete random variable. This formulation directly applies to mutation and SNP data as their information are intrinsically discrete. However, for features with numerical values (mRNA and microRNA expressions, copy number variations, DNA methylations), simple quantization with hard thresholds results in substantial information loss. To avoid this problem we adopted a *probabilistic quantization* procedure to convert a measurement outcome into a probabilistic vector over the discrete states. This procedure hypothesizes that the underlying molecular states are discrete with uncertainty and/or different mixture coefficients in a population of cells, and the uncertainties or mixture coefficients are reflected in the magnitudes of measurement outcomes. Therefore, information pertaining to magnitudes of measurement outcomes are preserved in the probability vectors of the discrete states.

Mutation data were directly converted into probability vectors by assigning the entire probability mass on the reported states. An entry without mutation (value 0) was converted into vector (Pr(no mutation)=1,Pr(mutation)=0). An entry with either point mutation (value 1) or nonsense mutation (value 2) was converted into vector (Pr(no mutation)=0,Pr(mutation)=1). An entry with a missing value was assigned an equal probability for each state (Pr(no mutation)=0.5,Pr(mutation)=0.5).

SNP data were treated analogously by converting into probability vectors of trinary states, whereas the entire probability mass was assigned to one of the three states. Each entry of the processed SNP data encodes the combinatorial state of two alleles – two homozygous states (e.g., AA or GG) and one heterozygous state (e.g., AG). This state space is much smaller than all possible combinations of dinucleotides (16) since at most two nucleotide variations appear on one locus with sufficiently high frequencies.

The data of mRNA, microRNA expressions and DNA methylations constitute continuous measurements from microarrays. We converted these continuous values into probability vectors of trinary states – up, down regulation or no change. For each dataset, denote z_{ij} the observed value of probe i on sample j, and x_{ij} its discrete hidden state. The following procedures convert each z_{ij} into a probability vector $(P(x_{ij} = -1), P(x_{ij} = 0), P(x_{ij} = 1))$.

1. If z_{ij} has a missing value, then assign equal probability $(P(x_{ij} = -1) = \frac{1}{3}, P(x_{ij} = 0) = \frac{1}{3}, P(x_{ij} = 1) = \frac{1}{3})$ to each state.

2. Rank-transform z_{ij} into the cumulative distribution function (CDF) value $y_{ij} \in [0, 1]$. For the datasets reporting relative values (Agilent arrays), rank transformation is applied to the entire matrix. For the datasets reporting absolute values (Affymetrix and microRNA data), each feature is rank-transformed separately. This is because we want to capture the relative variation of features across different samples instead of comparing the values of distinct features. DNA methylation data are scaled in $[0, 1]$ thus need not to be rank-transformed.

3. Convert y_{ij} into a probability vector $(P(x_{ij} = -1), P(x_{ij} = 0), P(x_{ij} = 1))$ with a specific quantization function. Intuitively, a data point with a low CDF value is more likely to be down-regulated ($P(x_{ij} = -1)$ is high), and a data point with a high CDF value is more likely to be up-regulated ($P(x_{ij} = 1)$ is high). This intuition is translated into the requirements that a quantization function is monotonic and maps $y_{ij} = 0$ into $P(x_{ij} = -1) = 1$

and $y_{ij} = 1$ into $P(x_{ij} = 1) = 1$. We chose polynomial functions f_γ and $\bar{f}_\gamma$ as the quantization curves.

$$\begin{aligned} P(x_{ij} = 1|y_{ij}, \gamma) &= f_\gamma(y_{ij}) \equiv y_{ij}^\gamma. \\ P(x_{ij} = -1|y_{ij}, \gamma) &= \bar{f}_\gamma(y_{ij}) \equiv (1 - y_{ij})^\gamma. \\ P(x_{ij} = 0|y_{ij}, \gamma) &= 1 - P(x_{ij} = 1|y_{ij}, \gamma) - P(x_{ij} = -1|y_{ij}, \gamma). \end{aligned} \tag{1}$$

Parameter γ controls the "soft thresholds" of assigning x_{ij} to be +1 or -1. A higher γ lifts the threshold on y_{ij} (and $1 - y_{ij}$) of calling the hidden state x_{ij} to be 1 (and -1). Thus a higher γ raises $P(x_{ij} = 0)$ and reduces $P(x_{ij} = \pm 1)$.

4. Quantization results are sensitive to γ values. To reduce the bias induced by a specific quantization function we assigned weights (prior) on f_γ functions and integrate the transformed values over a family of quantization functions. In this work we chose an exponential prior $e^{-(\gamma-1)}$ and restricted $\gamma \in [1, \infty)$. The averaged quantization outputs are:

$$\begin{aligned} P(x_{ij} = 1|y_{ij}) &= \int_1^\infty e^{-(\gamma-1)} f_\gamma(y_{ij}) d\gamma &&= \frac{y_{ij}}{1-\log y_{ij}}. \\ P(x_{ij} = -1|y_{ij}) &= \int_1^\infty e^{-(\gamma-1)} \bar{f}_\gamma(y_{ij}) d\gamma &&= \frac{1-y_{ij}}{1-\log(1-y_{ij})}. \\ P(x_{ij} = 0|y_{ij}) &= 1 - P(x_{ij} = 1|y_{ij}) - P(x_{ij} = -1|y_{ij}). \end{aligned} \tag{2}$$

The exponential prior $e^{-(\gamma-1)}$ was chosen for the following reasons. First, large γ values are penalized because they assign the probability mass to $x_{ij} = 0$ for most y_{ij} values. An exponential prior naturally penalizes large γ values. Second, it ensures the existence of the integrals in equation 2. Third, the requirements that $P(x_{ij} = 1|y_{ij} = 1) = 1$ and $P(x_{ij} = -1|y_{ij} = 0) = 1$ are satisfied. Fourth, the most justified single value of γ is $\hat{\gamma} = \frac{\log 3}{\log 2}$ because it assigns an equal probability ($\frac{1}{3}$) for each state when the input CDF $y_{ij} = 0.5$. The marginal quantization curves indeed resemble the quantization curves generated by $\hat{\gamma}$.

These procedures converted the measurement value of one probe in one sample into a probability vector. Genes are the elementary units in our analysis. The expression and DNA methylation of a gene are often measured by multiple probes in multiple datasets. The probe-level data were first rank-transformed into CDF values. For each dataset, we generated gene-level data by merging the probe data corresponding to the same genes. The entry of each gene in each sample was the average over all probe values of the corresponding gene and sample. The gene-based data from multiple sources were then further merged into one dataset. Likewise, entries of the same gene and sample from multiple sources were merged by taking an average. The CDF values of the merged datasets were then converted into probability vectors following steps 3 and 4 of the procedures.

CNV data processing

The aforementioned procedures of data normalization need to be modified on CNV data as the underlying assumption mismatches the empirical characteristics of CNV measurements. Equation 2 gives the probability of each trinary state x given the CDF value of its measurement outcome y. For each specific probe (gene), y is uniformly distributed in

$[0,1]$. Thus the total probability of encountering the up-regulation state ($x=1$) is

$$\begin{aligned} P(x=1) &= \int_0^1 P(y)P(x=1|y)dy \\ &= \int_0^1 \frac{y}{1-\log y}dy \\ &\approx 0.3663. \end{aligned} \tag{3}$$

Similarly, $P(x=-1) \approx 0.3663$ and $P(x=0) \approx 0.2674$. This distribution stipulates that the fractions of amplification/deletion/no change entries in the CNV data are approximately comparable. In reality, the vast majority of the CNV data entries do not deviate from normal values (0s for two-channel microarrays). Only about 1% of the data points have $\log_2$ ratios ≥ 1 or ≤ -1. Therefore, the quantization function in equation 2 severely distorts the global characteristic of the CGH array data.

To reduce this distortion we introduced an extra parameter β to the quantization function:

$$\begin{aligned} P(x_{ij}=1|y_{ij}) &= \frac{y_{ij}^{\beta}}{1-\log y_{ij}^{\beta}}. \\ P(x_{ij}=-1|y_{ij}) &= \frac{1-y_{ij}^{\beta}}{1-\log(1-y_{ij}^{\beta})}. \\ P(x_{ij}=0|y_{ij}) &= 1-P(x_{ij}=1|y_{ij})-P(x_{ij}=-1|y_{ij}). \end{aligned} \tag{4}$$

The new $P(x_{ij}=1|y_{ij})$ and $P(x_{ij}=-1|y_{ij})$ shrink with increasing β values. We adjusted β to make the global distribution $(P(x=1), P(x=0), P(x=-1))$ obtained from equation 4 close to the empirical distribution. For each CGH dataset, we counted the fractions of entries exceeding $\log_2(\frac{3}{2}) = 0.585$ (f_1) and below $\log_2(\frac{1}{2}) = -1$ (f_2). For simplicity we set the global empirical probability of amplification and deletion to be equal:

$$P(x=1) = P(x=-1) \approx \frac{1}{2}(f_1+f_2). \tag{5}$$

We then found the parameter value $\hat{\beta}$ that fit the following equality:

$$\begin{aligned} \int_0^1 \frac{x^{\hat{\beta}}}{1-\log x^{\hat{\beta}}}dx &\approx P(x=1). \\ \int_0^1 \frac{(1-x)^{\hat{\beta}}}{1-\log(1-x)^{\hat{\beta}}}dx &\approx P(x=-1). \end{aligned} \tag{6}$$

The estimated parameter $\hat{\beta}$ was substituted in equation 4 in probabilistic quantization. In this study, $\hat{\beta}$ is 37 for the CGH data generated by Harvard Medical School and 42 for the CGH data generated by Memorial Sloan-Kettering Cancer Center.

Unlike mRNA expressions or DNA methylations, the elementary subunits of CNV data are segments bounded by amplification and deletion events instead of genes. We introduce the following algorithm to partition each chromosome into segments with coherent CNV profiles and generate the representative CNV profiles for each segment.

First, we converted entries in each CGH dataset into trinary probability vectors using the rescaled probabilistic quantization in equation 4.

Second, on each dataset and each sample, we invoked a recursive algorithm to partition each chromosome into one or multiple segments. The CGH data of all probes on the same segment were treated as noisy instantiations of a common hidden variable (a naive Bayes model, [1]). Denote $S = \{\pi_1, \cdots, \pi_m\}$ a collection of m consecutive probes and X_S their normalized data. An entry $X_S(i, j, k) \in X_S$ denotes the probability that probe i is at state k on sample j. The log likelihood function $L_j(X_S)$ of a naive Bayes model of X_S on sample j is computed by the following procedures:

1. For each sample j evaluate the fractional counts for each state k: $f_j(k) = \sum_{i=1}^{m} X_S(i, j, k)$.
2. Choose the state $\hat{k}_j = \arg\max_k f_j(k)$ that maximizes the fractional count on sample j. Assign the state of the hidden variable on sample j to the consensus state over all probes: $y_S(j) = \hat{k}_j$.
3. For each sample j, calculate the transition probabilities of observing a probe state l conditioned on a hidden state k: $q_j(l|k) = \frac{I(y_S(j)=k) f_j(l)}{\sum_{l'=-1}^{1} I(y_S(j)=k) f_j(l')}$.
4. The log likelihood of the naive Bayes model is $L_j(X_S) = \sum_{k=-1}^{1} [N(y_S(j) = k) \log f_j(k) + \sum_{l=-1}^{1} (I(y_S(j) = k) f_j(l)) \log q_j(l|k)]$.

To partition a chromosome CNV on a specific sample j, we initially set S to be the probes of the entire chromosome and incurred the function *partition*(S):

1. Evaluate $L_j(X_S)$.
2. Find the binary partition (S_1, S_2) of S that maximizes $L_j(X_{S_1}) + L(X_{S_2})$.
3. Stop and return S if $L(X_S) \geq L(X_{S_1}) + L(X_{S_2}) - 5$.
4. Otherwise incur *partition*(S_1) and *partition*(S_2).

Third, since the CGH datasets have dense probes covering each chromosome (e.g., the CGH data generated by Harvard Medical School contains 10613 probes on chromosome 1), direct application of *partition*(S) to the entire chromosome is time and space consuming. Instead, we adopted a two-phase approach. In the first pass, we generated a low-resolution CNV data by sampling one out of every 1000 probes regularly. Recursive partitioning was applied to the low-resolution data and identified the break points where the chromosome was partitioned. In the second pass, recursive partitioning was applied to the probe windows surrounding the break points to obtain the refined boundaries of the segments.

Fourth, we aligned the partitioned segments generated by the two datasets. Since the two datasets probed the same collection of samples, consistent segments should emerge from both of them. We extracted the chromosome coordinates of partitioned segments from each dataset and constructed a bi-partite graph between the two sets of segments. Two segments were adjacent if their coordinates overlapped. Connected components of the bi-partite graph corresponded to aligned segment pairs. When there were multiple segments from each dataset in an aligned group, we disambiguated the alignment by finding the maximally matched segment pair. The results were consistent partitions from the two datasets.

Fifth, each sample may possess distinct CNV partitions as it encounters different copy number amplification/deletion events. The partition of the entire dataset is the union of all partitions across all samples. However, certain amplification and deletion events may recur in close but not identical positions. Ignoring these hotspots will result in many

small fragments. To avoid this problem we inspected the boundaries arising from each sample, merged spatially close boundaries and removed small fragments likely caused by mis-alignments.

Sixth, we took the union of the processed boundaries derived from all samples and created segments between consecutive boundaries.

Clustering DNA methylation, mRNA and microRNA expression data

Correlated effector aberrations tend to fit the same set of target genes. It is therefore more efficient to cluster the effector aberrations and build association modules with the proxy data derived from clustered effector aberrations. We propose a graph-based method to cluster DNA methylation, mRNA and microRNA expression data. In brief, by setting a threshold on correlation coefficients we could build a graph with genes as nodes and edges connecting correlated genes. By gradually lowering the threshold we extracted cliques – maximally and completely connected components – with increasing sizes. Highly connected cliques were then merged to form clusters.

1. Calculate the correlation coefficient matrix of the data.
2. Sort the pairwise correlation coefficients with a decreasing order.
3. Set the threshold to the highest correlation coefficient value.
4. Build a graph G with nodes as genes and edges connecting genes whose correlation coefficients exceed the threshold.
5. Find cliques on G.
6. Repeat the following steps until the threshold value ≤ 0.4.
   (a) Lower the threshold value. Add edges to G according to the new threshold value.
   (b) Find all existing clique pairs that become fully connected on the updated G. Among them merge the clique pair with the largest joint size.
   (c) Find the nodes that are connected to existing cliques with the new threshold value. Assign each node to the largest connecting clique.
   (d) Find newly emerged cliques with the new threshold value by incrementally adding nodes with high connectivity.
   (e) Update clique information.
7. Start with cliques as clusters, repeat the following steps until no clusters are mergeable:
   (a) For each pair of clusters, calculate the means of intra-cluster and inter-cluster correlation coefficients. Also calculate the mean of the joint cluster correlation coefficient.
   (b) Find the cluster pairs whose joint mean correlation coefficient ≥ 0.4 and the difference between the intra-cluster mean correlation coefficient and inter-cluster mean correlation coefficient ≤ 0.1.
   (c) Among the candidate cluster pairs merge the ones with the largest joint size.
   (d) Update the cluster information.

Construction of association modules

Figure 2 of the main text summarizes the procedures of constructing association modules from processed datasets. We describe each step in more details.

Pairwise associations

For each pair of molecular aberration (mutation, SNP, segment CNV, DNA methylation, microRNA expression) and mRNA gene expression, we evaluated their Pearson correlation coefficient, log-likelihood ratio of the logistic regression model, and p-value obtained from both χ^2 approximation of log-likelihood ratio and permutation tests. Denote x a candidate effector and y a target gene expression. We express the conditional probability $P(y|x)$ with a logistic regression model:

$$P(y|x) = \frac{1}{Z(x)} e^{\lambda f(x) y}, \lambda \geq 0. \tag{7}$$

where either $f(x) = x$ (the effector activates the target) or $f(x) = -x$ (the effector represses the target). λ is a nonnegative parameter. $Z(x) = 1 + e^{\lambda} + e^{-\lambda}$ is the partition function that normalizes the conditional probabilities.

Denote $D \equiv (x^1, y^1), \cdots, (x^m, y^m)$ the observed instantiations of x and y over m samples. The log-likelihood function of the observed data D is

$$\begin{aligned} L(D;\lambda) &= \sum_{k=1}^{m} \{\log(P(x^k)) + \log(\tfrac{1}{Z(x)}) + \lambda f(x^k) y^k\} \\ &= \sum_{C_x, C_y} \{N(C_x) \log(P(C_x)) + N(C_x, C_y)[-\log(Z(C_x)) + \lambda f(C_x) C_y]\}. \end{aligned} \tag{8}$$

where C_x and C_y stand for configurations of x and y, and $N(C_x), N(C_x, C_y)$ the fractional counts for configurations C_x and (C_x, C_y) over all samples. Using probabilistic quantization each entry x_{ij} was converted into $(P(x_{ij} = -1), P(x_{ij} = 0), P(x_{ij} = 1))$, where i and j are gene and sample indices. The fractional count for a state configuration (C_x, C_y) is

$$N(C_x, C_y) = \sum_j P(y_j = C_y) P(x_j = C_x). \tag{9}$$

The maximum likelihood parameter $\hat{\lambda}$ was numerically estimated using the Newton-Raphson method. Denote $F(\lambda) \equiv L'(D; \lambda)$ and $G(\lambda) \equiv F'(\lambda)$. Set $\lambda^0 = 1$ as the initial value of λ. Iteratively execute the following updates until either λ^t converges or $\lambda^t \leq 0$:

$$\lambda^{t+1} = \lambda^t - G^{-1}(\lambda^t) F(\lambda^t). \tag{10}$$

To test the significance of the pairwise association, we introduced a null model M_0 where x and y are independent ($\lambda = 0$) and treated equation 7 as an alternative model M_1. The log-likelihood $L(D; M_1)$ was given by equation 8. The log-likelihood $L(D; M_0)$ was simply $-m \log N_x$, where N_x denotes the number of states of variable x.

Given the observed data D and two nested models $M_0, M_1 \supseteq M_0$, we incurred a standard hypothesis testing procedure to calculate the log-likelihood ratio and χ^2 p-value:

$$\begin{aligned} \mathcal{L}(D; M_0, M_1) &= L(D|M_1) - L(D|M_0). \\ p &= 1 - \chi_1^2(2\mathcal{L}(D; M_0, M_1)). \end{aligned} \tag{11}$$

where χ_1^2 is the χ^2 CDF function with one degree of freedom.

The χ^2 p-values tend to over-estimate the significance of the testing results. Thus we also evaluated the p-values of permutation tests and reported the supremum of χ^2 and permutation p-values. Permutation p-values were calculated by the following procedures:

1. Quantize the aberration and expression CDF values into binary or trinary states. For trinary variables we choose 0.4 and 0.6 as thresholds.
2. Count the number of samples where the aberration and expression states are consistent with the truth tables from M_1. Denote this number as n_C.
3. Find the additional feature variables X_v that appear in M_1 but not in M_0.
4. Repeat the following steps 10000 times:
   (a) Randomly permute the data in X_v.
   (b) Count the number n_P of samples where the permuted aberration and expression states are consistent with M_1.
5. The p-value is the fraction of n_P's exceeding n_C.

The reported p-value for each pairwise association is the maximum of the p-values derived from χ^2 approximation and permutations.

The outputs of pairwise associations were filtered with the following thresholds: absolute values of correlation coefficients ≥ 0.2, log-likelihood ≥ 1.0, p-values ≤ 0.1. The purpose of imposing these loose thresholds is to discard the enormous number of weak pairwise associations. Only the pairs passing all the three thresholds were reported as candidates for the association models.

Building association models for individual genes

Not all types of molecular aberrations are equally likely to drive gene expressions. Some candidate effectors provide direct explanations for gene expressions without requiring many mechanistic assumptions underlying gene regulation (e.g., cis-acting effects with CNVs). Others have massive number of features thus are likely to introduce spurious associations (e.g., SNPs). We proposed a *layered modeling framework* to prioritize molecular aberrations differently and incrementally incorporating candidate effectors to the model according to their priorities. Molecular aberrations are categorized with the following priorities.

1. Level 1: segment CNVs on the same chromosome as the target (CNV cis-acting effects), mutations and DNA methylations on the target, SNPs on the same chromosome with the target.

2. Level 2: positive associations with segment CNVs on distinct chromosomes from the target (CNV trans-acting effects), mutations of non-target genes, negative associations with DNA methylations of non-target genes, non-local SNPs.
3. Level 3: negative associations with CNV trans-acting effects when the target is not explained by level 1-2 models.
4. Level 4: negative associations with microRNA expressions when the target is not explained by level 1-3 models.

In addition, we placed an extra requirement for CNV trans-acting effects. To build an association between a segment CNV and a target gene on another chromosome, the segment must accommodate at least one intermediate regulator that likely modulates the expression of the target. The regulator gene expressions are positively associated with the segment CNVs, and the functional direction of the association between the regulator and the target gene expressions coincides with that of the association between the segment CNV and the target gene expression. 727 candidate regulators were pulled from three sources: human transcription factors from TRANSFAC [2], human transcription factors and signaling proteins from FanTom [4], and genes pertaining to cancer from the OMIM database [3].

Pairwise associations were filtered with different thresholds of log-likelihood ratios, p-values and correlation coefficients according to the levels and types of effectors. The thresholds applied in the analysis are reported in Supplementary Table S4.

We again applied logistic regressions to build association models with multiple effectors. The procedures of calculating the maximum log-likelihood of a model are analogous to those for pairwise associations (equations 7-10).

The log-likelihood ratio, χ^2 p-value and permutation p-value between two nested models $M_0, M_1 \supseteq M_0$ were calculated analogous to equation 11. Here the degree of freedom d of the χ^2 function χ^2_d is the number of additional features in M_1 compared to M_0.

The hypothesis testing procedures were applied in a model selection algorithm as follows. Suppose M_0 is a null model containing no effectors, M_1 is an association model at the current level. Consider adding a candidate effector x to the current association model M_1. We define the following model selection procedures as $selection(M_1, x, \theta, \theta')$:

1. Build a model M_2 containing a single effector x, and a model $M_{12} \supset M_1$ by adding an additional term of x to the logit of M_1.
2. Incur three hypothesis tests: M_2 against M_0, M_{12} against M_1, and M_{12} against M_2. Denote their p-values as p_0, p_1, p_2 respectively. The following operations are executed depending on the testing outcomes:
   (a) If $p_0 > \theta$, then discard x since its association with the target is weak.
   (b) If both $p_1 \leq \theta'$ and $p_2 \leq \theta'$, then the joint model M_{12} provides an additional explanatory power relative to both M_1 and M_2. Replace M_1 with M_{12}.
   (c) If $p_1 \leq \theta'$ and $p_2 > \theta'$, then the joint model M_{12} is better than M_1 but not M_2. This implies that the explanatory power of M_1 is dominated by M_2 but not the other way around. Replace M_1 with M_2.
   (d) If $p_2 \leq \theta'$ and $p_1 > \theta'$, then the joint model M_{12} is better than M_2 but not M_1. This implies that the explanatory power of M_2 is dominated by M_1 but not the other way around. Discard x.

(e) If both $p_1 > \theta'$ and $p_2 > \theta'$, then neither M_1 nor M_2 can dominate the other, and the joint model does not provide an additional explanatory power. Keep M_1 and add M_2 to the current model list, yet do not merge M_1 and M_2.

θ and θ' stand for the p-value thresholds for testing a single-effector model (M_2) against an independent model (M_0) and testing a multi-effector model (M_{12}) against a null model by removing one effector (M_1 or M_2). The test is significant if the p-value is not greater than the threshold. In this study, we chose θ and θ' according to Supplementary Table S4.

To build association models to explain a gene expression data y, we employed the layered modeling framework to incrementally add effector molecular aberrations to the existing models with the following procedures. Initially set the association model $\mathcal{M}$ as empty.

1. At level 1, implement the following steps.
   (a) Filter out candidate effectors according to the thresholds of log-likelihood ratios, p-values and correlation coefficients listed in Supplementary Table S4.
   (b) Among all the CNV segments located on the same chromosome of y, keep the one with the highest log-likelihood ratio.
   (c) For each remaining candidate effector x at level 1, apply $selection(M, x, \theta, \theta')$ to each current model $M \in \mathcal{M}$. The effectors are incorporated according to the following order: CNV cis-acting effects, mutations, DNA methylations.
2. At level 2, implement the following steps.
   (a) Filter out candidate effectors according to the thresholds of log-likelihood ratios, p-values and correlation coefficients listed in Supplementary Table S4.
   (b) Remove the CNV positive trans-acting associations that do not contain candidate regulators with consistent associations with both segment CNVs and target gene expressions.
   (c) If multiple CNV segments on the same chromosome have trans-acting effects with y, then only keep the one with the strongest log-likelihood ratio.
   (d) For each remaining candidate effector x at level 2, apply $selection(M, x, \theta, \theta')$ to each current model $M \in \mathcal{M}$. The effectors are incorporated according to the following order: CNV trans-acting effects, SNPs, mutations, DNA methylations.
   (e) Multiple effector candidates of DNA methylations may possess significant explanatory power for they share correlated methylation profiles. To prevent including all these correlated DNA methylation effectors in the model selection process, we solicited one representative from each cluster of DNA methylation data. The representative DNA methylation profile possesses the highest log-likelihood ratio among the members in the same cluster. When incorporating higher-layered associations, only the representative DNA methylations will be included in the association model of the current level. Non-representative DNA methylations that pass the model selection procedures will be reported as effectors but not used in evaluating log-likelihood ratios. Notice model selection still operates at the DNA methylation profiles of single genes, and non-representative

DNA methylations are still included as effectors. Clustering results are used only to reduce model complexity in model selection.

3. At level 3, implement the following steps.
   (a) Filter out candidate effectors according to the thresholds of log-likelihood ratios, p-values and correlation coefficients listed in Supplementary Table S4.
   (b) Remove the CNV negative trans-acting associations that do not contain candidate regulators with consistent associations with both segment CNVs and target gene expressions.
   (c) If multiple CNV segments on the same chromosome have trans-acting effects with y, then only keep the one with the strongest log-likelihood ratio.
   (d) For each remaining candidate effector x at level 3, apply $selection(M, x, \theta, \theta')$ to each current model $M \in \mathcal{M}$.
4. At level 4, implement the following steps.
   (a) Filter out candidate effectors according to the thresholds of log-likelihood ratios, p-values and correlation coefficients listed in Supplementary Table S4.
   (b) For each remaining candidate effector x at level 4, apply $selection(M, x, \theta, \theta')$ to each current model $M \in \mathcal{M}$.
   (c) Similar to DNA methylations, many microRNA expression profiles are highly correlated. To avoid including these correlated microRNAs into the model we solicited one representative from each cluster and included only the representative microRNAs in the model selection process.

The outcome is a list of association models that explain the mRNA expression of each gene.

Assembling association models of individual genes to modules

Molecular aberrations on effector genes or genomic components often mis-regulate many downstream targets. We define an association module as a tuple consisting of three components: (1)observed effector molecular aberrations, (2)target genes, (3)regulators that mediate the effects between effectors and targets. From the association models of individual genes we incurred the following procedures to construct modules.

1. Group gene expressions by each candidate effector molecular aberration. Assign a gene to the targets of a effector molecular aberration if the latter appears in the association model(s) of the former.
2. Merge all the CNV cis-acting modules with segments on the same chromosome together. The effectors and targets are the union of those in all the member modules. Hence there is at most one CNV cis-acting module per chromosome.
3. Merge all the CNV trans-acting modules of identical functional direction and with segments on the same chromosome together. Merge intermediate regulators in addition to targets and targets. There are at most two CNV trans-acting modules per chromosome (one positive and one negative association).
4. Discard small modules with less than 20 target genes.
5. Construct an undirected graph GM of *mergeability* among the remaining modules according to the following criteria.

(a) Do not merge modules with CNV cis-acting effects.

(b) Do not merge module pairs with CNV trans-acting effects.

(c) If the intersection of targets between two modules exceeds one third of the target set of the smaller module, then the two modules are mergeable and add an edge connecting them in GM.

(d) If the intersection of targets between two modules exceeds 50 genes, then add an edge connecting the two modules in GM.

6. Find connected components in GM. Merge the modules in each connected component together.

In-silico validation of association modules

FDR evaluation

False discovery rates (FDRs) quantify the expected fraction of false positives among the positive calls from a multiple-hypothesis testing problem. To simplify the testing procedures, we considered a positive call as a significant pairwise association between a effector and a target. The thresholds of log-likelihood ratios, p-values and correlation coefficients for determining positive calls are reported in Supplementary Table S4. Significant pairwise associations arising from the permuted data are false positives according to the null model. Lacking specific information about the distribution of noise, we created a simple null model by randomly permuting the effector aberration and target expression 1000 times. Such permutation tests preserve the marginal distributions of individual components thus are widely used in evaluating the significance of detected signals. The empirical distribution of the number of significant pairwise associations arising from permuted data provides a reasonable measure for false positive numbers. A standard formula of FDR is $\frac{E\{\#\text{ false positives according to the null model}\}}{\#\text{ positive calls from the data}}$ [6]. The expected number of false positives can be directly calculated using its distribution from the permuted data. This quantity, however, may over-estimate accuracy when more false positives arise with a non-negligible probability. Alternatively, we also located a false positive number in the tail of its distribution and evaluated $\frac{\#\text{ false positives in the 99 percentile of the null model}}{\#\text{ positive calls from the data}}$ [7]. The second quantity is a much more conservative measure of FDR. Hence the reported pairwise associations are more confident if their revised FDR value is small. We evaluated the two FDRs for each type of associations (CNV cis-acting effects, CNV trans-acting effects, mutations, DNA methylations, microRNA expressions) separately. The aggregate FDRs were evaluated by the distribution of false positive numbers arising from all types of associations together.

Enrichment analysis of functional categories and pathways

We extracted 3312 categories from the Gene Ontology (GO, [9]) database according to the following criteria: (1)categories are not subcellular locations, (2)they contain at least 10 genes appeared in the TCGA datasets, (3)they contain less than 1000 genes. Condition 1 excludes the categories irrelevant to gene functions. Condition 2 avoids small categories with marginal overlap with the TCGA data. Condition 3 discards the large and generic categories like “metabolism” or “protein binding”. Analogously, 889 pathways were extracted from three separate sources: Reactome [10], BioCarta [11], and the NCI Pathway Interaction Database [12]. We treated a pathway as a collection of genes without considering its topology.

Two additional collections of gene sets were incorporated in enrichment analysis. A panel of 35 genes were divided them into 3 subclasses: protoneural, mesenchymal and proliferative [13]. Glioma patients with up-regulated genes in each subclass have disparate survival durations. Ben-Porath et al. constructed 13 gene sets associated with embryonic stem (ES) cell identity and found that poorly differentiated tumors showed preferential overexpressions of the ES-associated genes [14].

For each association module and functional gene set, we evaluated the enrichment significance with a Fisher's exact test. Denote N, n and m as the total number of genes, the number of target genes in the module, and the size of the functional gene set respectively. Suppose the module targets and the functional gene set overlap in k genes. The hypergeometric p-value assesses the probability of observing $\geq k$ overlapped genes when randomly selecting n genes from the sample:

$$p = \sum_{k'=k}^{min(n,m)} \frac{\binom{m}{k'}\binom{N-m}{n-k'}}{\binom{N}{n}}. \tag{12}$$

The denominator $\binom{N}{n}$ enumerates possible combinations of n-gene subsets. The numerator $\binom{m}{k'}\binom{N-m}{n-k'}$ enumerates possible combinations of choosing k' genes from the functional set and $n-k'$ genes from its complement. Summation is carried over $k' \geq k$.

The probability of finding spurious enrichment increases with the number of functional gene sets considered. We adjusted the p-values of multiple hypotheses tests with a Bonferroni correction: the adjusted p-values are the hypergeometric p-values multiplying the number of GO categories (3312), pathways (889), glioma subclasses (3), and ES-like gene sets (13) considered.

Co-citation analysis on PubMed database

We incurred a batch search on the PubMed database to find all the pairs of effectors/regulators and targets in each module that were co-cited in the same publications. Overall, 676 effector (regulator)-target pairs are co-cited in previous publications.

To assess the significance of co-citations in each module, we generated all possible pairs between 92 effectors/regulators and 22697 genes in the TCGA dataset, and incurred a batch search on PubMed to find all the genes co-cited with each effector/regulator. For each effector/regulator, the background (null) model is to randomly draw genes from 22697 members. The p-value of enrichment of co-cited targets in a module is approximated by a binomial distribution:

$$p = \sum_{k'=k}^{n} \binom{n}{k'} q^{k'} (1-q)^{n-k'}. \tag{13}$$

where q denotes the fraction of genes co-cited with the effector/regulator, n and k denote the numbers of targets and co-cited targets in a module respectively.

Reproducibility verification of associations in external datasets

We verified the reproducibility of the association modules by checking whether the associations extracted from each module are robust in external datasets containing GBMs and other CNS tumors. Supplementary Table S3 lists the information of the 8 external datasets of gene expressions. For each module, we incurred the following three tests to verify the reproducibility of associations.

1. *Coherence of target gene expressions.* We computed the distributions of correlation coefficients between the target gene expressions in all the 9 datasets (including the TCGA data). To quantify the strength of coherence, we compared the distribution of correlation coefficients with the background distribution derived from a reference set of genes. A natural choice for the reference set is all the genes in the dataset. This choice, however, is too relaxed as the global distribution of expression correlation coefficients is sharply concentrated around 0 and any small deviation yields significant p-values. Instead, we chose the intersection of TCGA genes and members in GO category of ribosomes (accession number 0005840) as the reference set. Ribosome genes were strongly co-expressed across many distinct datasets. Therefore, the coherence of targets is more confident if it stronger than that of the ribosome genes. We evaluated the p-value of the one-sided, two-sample Kolmogorov-Smirnov test between the two distributions of correlation coefficients.
2. *Associations between effectors and targets.* To verify the reproducibility of association modules it is also necessary to demonstrate that the associations between effector molecular aberrations and target gene expressions are preserved in external datasets. Since molecular aberration data (CNVs, mutations, DNA methylations) were not available in the external datasets, we had to find proxies of molecular aberrations in the gene expression data. For mutations and DNA methylations, the proxies were simply the expressions of the mutated/methylated genes. These choices hypothesize that alterations on DNA sequences or epigenetic states of a gene modulate its own expression. For CNVs on a chromosome, we chose the expressions of its constituent genes belonging to the targets of its CNV cis-acting module as the proxies. Targets in the CNV cis-acting modules are likely modulated by the amplifications and deletions of chromosomes, thus are sensible proxies for chromosome CNVs. For each module, we calculated the distribution of correlation coefficients between effector proxies and target expressions. As a comparison, we evaluated the distribution of correlation coefficients between effector proxies and expressions of all genes in the dataset. Unlike the test for target coherence, ribosome genes are not an adequate reference set since no evidence supports strong associations between effector proxy expressions and ribosome gene expressions. The p-values of the two sample, one-sided Kolmogorov-Smirnov tests between the two distributions were reported.
3. *Associations between regulators and targets.* For modules with CNV trans-acting effects, there exist intermediate regulators associated with both effector molecular aberrations and target gene expressions. Thus it is natural to check whether regulator and target gene expressions are associated in external datasets. We calculated the distributions of expression correlation coefficients between regulators and targets and between regulators and all the genes in the dataset. The p-values of the two sample, one-sided Kolmogorov-Smirnov tests between the two distributions were reported.

Inferring recurrent molecular aberrations in GBMs

We postulate that certain molecular aberrations are specific in GBMs and modulate their constituent gene expressions. Consequently, these GBM-specific molecular aberrations should manifest disparate gene expressions between GBM and non-GBM samples.

To uncover these GBM-specific molecular aberrations, it is necessary to analyze the expression datasets containing considerable numbers of both GBM and non-GBM samples. By examining the 8 external datasets, we found only two of them contained at least 25 GBM and non-GBM samples: GSE16011 and GSE4412. Thus we used these two datasets to identify GBM-specific molecular aberrations.

For each chromosome CNV, we extracted the targets in its cis-acting module and calculated the distributions of their expression levels among GBM and non-GBM samples. Two sample, two-sided Kolmogorov-Smirnov tests between the two distributions were applied to check whether subset (say GBM samples) had consistently higher expression levels than another subset (say non-GBM samples) in both datasets. For each mutated or methylated gene, we compared the distributions of its expression levels between GBM and non-GBM samples using two-sided Kolmogorov-Smirnov tests.

Assessment of the prognostic power of association modules

The prognostic power of biomarkers is typically measured by Cox regression coefficients. In survival analysis, Cox regression coefficients quantify the association of a set of independent variables (e.g., biomarker gene expression levels) with the hazard function of the population [15].

We treated the mRNA expression of each gene as an independent variable in the Cox regression model and evaluated its regression coefficient. To assess the prognostic power of an association module, we evaluated the distribution of Cox regression coefficients of its targets and compared this distribution with the background distribution of all genes. Two-sided, two-sample Kolmogorov-Smirnov tests were applied to calculate the p-values. Deviations from the background distribution in both directions provide useful prognostic information. Strong positive coefficients indicate negative associations with survival durations (higher expression levels are associated with shorter survival durations), and strong negative coefficients indicate positive associations with survival durations (higher expression levels are associated with longer survival durations).

We selected datasets for survival analysis according to the following criteria: (1)patient survival information were available, (2)exact survival durations (in terms of days or weeks) instead of broad ranges were labeled, (3)there were at least 50 samples. The TCGA GBM and three other external datasets of CNS tumors satisfied these conditions: GSE16011, GSE4412 and GSE7696. In each dataset, we reported the p-values of Cox regression coefficient distributions of each module. Moreover, to demonstrate that the prognostic power of association modules is independent of tumor types and grades, we partitioned samples from GSE16011 into five subgroups – OD (oligodendroglial tumors) grade III (44 samples), GBM grade VI (156 sammples), A (astrocytomas) grade II (13 samples), A grade III (16 samples), and others (40 samples) – and evaluated the prognostic power of association modules on each subgroup.

Supplementary analysis results

SNP-mRNA associations

The genotype data of 850432 SNPs are reported. Due to the large number of SNPs considered we imposed a stringent threshold on filtering pairwise associations between genotypes and mRNA expressions (log likelihood ratio ≥ 7.0, p-value $\leq 10^{-5}$). Only 15 cis-acting SNP-mRNA pairs pass the threshold. In all the 15 pairs the SNP loci are within 50 kb from the mRNA genes. Twelve associations are additive (samples carrying two homozygotic alleles have high and low expression levels and samples carrying the heterozygotic allele have intermediate expression levels). Two pairs – rs4103004-*EFHB* and rs1993530-*ZNF880* – are congruent with dominant effects – samples carrying one homozygotic allele (e.g., AA) have low expression levels, and samples carrying another homozygotic (say CC) and heterozygotic (e.g., AC) alleles have high expression levels). Curiously, the rs17172433-*EGFR* pair exhibits a synergistic pattern – samples carrying the heterozygotic allele (e.g., AC) have lower expression levels than samples carrying either one of the homozygotic alleles (e.g., AA and CC). Supplementary Figure S4 visualizes the information of these 15 cis-acting eQTL pairs. Due to the small number of significant SNP-mRNA pairs, the cis and trans-acting effects with SNPs are not incorporated in the association modules.

Prognostic power of association modules

Despite the generally acuteness of GBMs, patients in the TCGA dataset possess very heterogeneous survival times ranging from a few weeks to 10 years. It is thus of great interest to identify the molecular aberrations predicting the prognostic outcomes. We extracted 4 expression datasets of CNS tumors (TCGA, GSE16011, GSE4412, GSE7696) and assessed the prognostic power of each gene expression profile by calculating its Cox regression coefficient and p-value with respect to survival times. The prognostic power of an association module is evaluated by comparing the Cox regression coefficient distribution of its targets with the background distribution of all genes.

Among the 45 association modules, 9 exhibit significant prognostic power (p-value $\leq 10^{-8}$) in at least 3 datasets. Supplementary Table S6 reports the prognostic power of all association modules in each dataset. The patient survival patterns are categorized into two groups. In modules 2, 11, 29, 41, 42 and 43, the Cox regression coefficients deviate from the background distributions in the positive direction. In modules 4 and 14, the Cox regression coefficients deviate from the background distributions in the negative direction. Module 1 manifests negative Cox regression coefficients in GSE7696 but has a positive tendency in the remaining 3 datasets.

Among the 4 CNS datasets the Erasmus data (GSE16011) provide information about tumor types and grades of samples. We demonstrate that the prognostic power of the 9 selected modules is independent of tumor types and grades in the Erasmus data. Supplementary Figure S6 displays the Cox regression coefficient distributions of the 9 selected modules in 5 sample subgroups of the Erasmsus data with distinct tumor types and grades. Clearly, the target Cox regression coefficients of all the 9 modules significantly deviate from the background distribution in the majority of sample groups. Among the 9 modules, 7 of them exhibits significant prognostic power (p-value $\leq 10^{-4}$) in at least four of five subgroups. The remaining two modules – modules 42 and 43 – have significant prognostic

power in three subgroups. A lower p-value threshold is chosen (10^{-4}) since the sample size of each subgroup is much smaller than the total sample size.

The directions of Cox regression coefficients are largely consistent with the known effects of effector molecular aberrations. A positive Cox regression coefficient indicates a negative association of the feature values with survival times. Chromosome 7 amplifications (modules 11 and 29) and *NF1* nonsense mutations (module 41) are associated with poor prognosis, as the patients with high target expression values tend to have shorter survival times. Conversely, chromosome 10 deletions (module 14) are associated with poor prognosis as patients with low target expression values tend to have shorter survival times. In addition to these well-known cases, there are also significant positive associations of survival times with *CD40* methylations (module 42), *GSTM1* methylations (module 43), *ZMYND10*, *RBP1* and *FES* methylations (module 2), and negative associations with chromosome 11 amplifications (modules 2), *PLXNB1* methylations and microRNAs *mir-21* and *mir-22* expressions (module 4).

Beyond the general directions of associations with survival times, we also evaluated the strength of prognostic predictions for each module by computing the Cox regression coefficient p-values of the median expression profiles among their targets and report the results in Supplementary Table S6. Modules 2 and 14 yield consistent and significant associations with survival times ($p < 0.1$) in all 4 datasets. Figure 3 of the main text visualizes the target expressions in modules 2 and 14 in relation to patients' survival times and the Kaplan-Meier curves of patients segregated by the median target expression values. In TCGA data, the effector molecular aberration levels (chromosomes 11 and 10 CNVs) and their corresponding Kaplan-Meier curves are also displayed. The targets in both modules retain coherent expressions in all the 4 datasets. Furthermore, module 2 effector CNV and target expression levels have strong negative associations with survival times, whereas module 14 effector CNV and target expression levels have strong positive associations with survival times.

The consistent and significant associations of modules 2 and 14 with survival times are also independent of tumor types and grades. Supplementary Figure S7 shows the Kaplan-Meier curves of patients segregated by the median target expression values in the 5 tumor subgroups from the Erasmus data. The p-values of the Cox regression coefficients are significant (p-value ≤ 0.1) in all tumor subgroups except grade II astrocytomas (row 4). The high p-values in grade II astrocytomas is likely due to their small sample size (13 samples).

Additional information strongly supports the prognostic relevance of modules 2 and 14. Module 2 harbors 7 of 11 genes involved in epithelial-mesenchymal transition according to [13]. Patients with high expressions of these genes are previously reported to suffer from poor prognosis. We examined the Cox regression coefficients of these genes in each CNS dataset and found they were all positive and largely significant except in GSE7696 (Supplementary Table S7). Furthermore, we identify 20 genes whose expression profiles yield consistent (Cox regression coefficients have identical directions) and highly significant (Cox regression p-values ≤ 0.01) prognosis in all 4 datasets. 16 of the 20 biomarkers belong to at least one module. In particular, module 2 consists of 6 biomarkers (*PLA2G2A*, *TMEM22*, *TBC1D1*, *NR2E1*, *KLHL26*, *RGN*), and module 14 consists of 7 biomarkers (*GTPBP4*, *PITRM1*, *ASB13*, *KIN*, *TFAM*, *WAPAL*, *BCCIP*).

The trans-acting module of chromosome 10 CNVs (module 1) tends to have negative Cox regression coefficients in GSE7696 and positive Cox regression coefficients in the remaining 3 datasets (Figure 3 of the main text). Paradoxically, the directions of Cox regression coefficients in module 1 are the opposite of the directions in the cis-acting module of chromosome 10 CNVs (module 14).

Alignment with GBM subtypes characterized by transcription and methylation profiles

Two prior studies have provided comprehensive and robust molecular classifications of GBMs. Verhaak et al. used a panel of 840 gene expression profiles to divide GBM tumors into four classes: proneural, neural, classical, and mesenchymal [16]. Noushmehr et al. found that a distinct subset of GBM samples displays concerted hyper-methylation at a large number of loci and named them as "glioma-CpG island methylator phenotype" (G-CIMP) [17]. To relate these classifications with the information derived from association modules, we classified tumor samples according to the median expressions of target genes in each module and counted the overlap of these binary classification outcomes with the subtypes from the two prior studies.

There are 63 classical subtype samples, 43 neural subtype samples, 62 proneural subtype samples and 74 mesenchymal subtype samples in the TCGA GBM data. For each module, we calculated the median expression profile over the target genes and used this expression profile as a "biomarker" of the module to assign tumors into binary classes. The threshold for binary classification was chosen to maximize the mutual information between the transcriptional subtypes and the binary class labels obtained from the biomarker expression values. We then counted the overlap between the two classification outcomes and reported the summary information in Figure 5 of the main text. No binary classifications based on median module target gene expressions can perfectly divide the samples into two groups consistent with the four transcriptional subtypes. However, a number of the module expression biomarkers can assign nearly all samples from at least one subtype to the same binary label. We define samples in a transcriptional subtype are concentrated in one binary class if no more than 10 samples fall in the complementary binary class. Moreover, we define the binary classification based on median module target gene expressions aligned with the subtype labels if at least one subtype is concentrated in each of the two binary classes. According to these criteria, 12 association modules are aligned with the transcriptional subtypes: 1, 2, 4, 29, 30, 31, 32, 33, 35, 40, 41 and 43. For instance, the overlap counts of module 1 are as follows: classical: 60 low biomarker expression and 3 high biomarker expression, neural: 13 low biomarker expression and 30 high biomarker expression, proneural: 42 low biomarker expression and 20 high biomarker expression, mesenchymal: 3 low biomarker expression and 71 high biomarker expression.

There are 21 G-CIMP positive samples and 224 G-CIMP negative samples in the TCGA data. For each association module, we evaluated the median target gene expression profile and constructed a binary classifier accordingly with the threshold 0.5. We counted the overlap of G-CIMP subgroups with the binary classes derived from each association module and reported the outcomes in Figure 5 of the main text. Similar to transcriptional subtypes, the G-CIMP positive samples are concentrated in one binary class derived from the median expressions of some association modules. In 16 of 45 modules, no less than 19 G-CIMP positive samples have concerted high or low median target

gene expressions. The G-CIMP positive samples exhibit high expression levels in 6 modules – 4, 12, 14, 34, 36, 39, and low expression levels in 10 modules – 2, 11, 29, 31, 32, 33, 40, 41, 42, 43. In contrast, G-CIMP negative samples are far more abundant thus are not concentrated in any binary class derived from the association modules.

Since the G-CIMP phenotypes have strong predictive power for prognosis and the target gene expressions of some modules are aligned with the G-CIMP status, the strong associations of module target gene expressions with survival times can be explained away by G-CIMP phenotypes. We evaluated the deviation of the Cox regression coefficient distributions between the module target genes and the entire gene set among the G-CIMP positive and G-CIMP negative samples separately. Supplementary Figure S8 shows the distributions of Cox regression coefficients on the modules with significant deviation (KS test p-value $\leq 10^{-6}$. Among the 9 association modules exhibiting strong prognostic power across multiple glioma datasets, module 1 retains significant associations with consistent directions in both G-CIMP positive and negative samples. Modules 2, 4 and 42 demonstrate significant associations with opposite directions in G-CIMP positive and negative samples. Modules 2 and 42 target gene expressions tend to have positive Cox regression coefficients (negative associations with survival times) in G-CIMP positive samples and negative Cox regression coefficients in G-CIMP negative samples. In contrast, module 4 tends to have negative Cox regression coefficients in G-CIMP positive samples and positive Cox regression coefficients in G-CIMP negative samples. The remaining 5 modules (11, 14, 29, 41, 43) possess significant associations in at most one G-CIMP subclass.

Prognostic power of the joint hallmark derived from the combination of multiple association modules

The prognostic power of most single association modules is explained away by G-CIMP phenotypes: conditioned on the G-CIMP phenotypes, the deviation of the Cox regression coefficients between the module targets and the reference set becomes insignificant (Supplementary Figure S8). However, since G-CIMP positive tumors harbor multiple favorable molecular characteristics, it is sensible to postulate that patients possessing concurrent and favorable molecular characteristics have superior prognostic outcomes regardless of the G-CIMP phenotype. To justify this postulation we considered the samples exhibiting down-regulation in the median target gene expressions of 10 modules – 2, 11, 29, 31, 32, 33, 40, 41, 42, 43, and up-regulation in the median target gene expressions of 6 modules – 4, 12, 14, 34, 36, 39. The threshold for determining up or down-regulation of an expression level was set to 0.5. 21 samples possess this joint hallmark, 14 and 7 belong to the G-CIMP positive and G-CIMP negative phenotypes respectively. We compared the Kaplan-Meier curves of four subclasses and displayed them in Supplementary Figure S9: G-CIMP positive with the joint hallmark (14 samples), G-CIMP positive without the joint hallmark (7 samples), G-CIMP negative with the joint hallmark (7 samples), G-CIMP negative without the joint hallmark (217 samples). Intriguingly, the survival times of G-CIMP negative patients with the joint hallmark are significantly longer than the G-CIMP patients without the joint hallmark (blue and red curves in Supplementary Figure S9, logrank p-value 0.03557). In contrast, there is no significant survival difference between the G-CIMP positive samples with and without the joint hallmark (black and magenta curves in Supplementary Figure S9, logrank p-value 0.5776). G-CIMP negative patients with the joint hallmark have inferior prognosis to G-CIMP positive patients but are superior to G-CIMP negative patients without

the joint hallmark. This ordering suggests that the joint hallmark provides additional information about prognosis relative to G-CIMP phenotypes.

To further reduce the number of necessary modules constituting the joint hallmark, we started with the joint hallmark of 16 modules and iteratively removed the modules without sacrificing its prognostic power. In each iteration, we removed the module such that the logrank p-value of the joint hallmark from the remaining modules was minimized. Iterations stopped when the logrank p-value among the G-CIMP negative population became ≥ 0.1. The trimmed joint hallmark consists of two down-regulated modules (11 and 42) and two up-regulated modules (12 and 14). 10 G-CIMP negative samples possess this joint hallmark. Supplementary Figure S10 displays the Kaplan-Meier curves of the four subclasses derived from the joint hallmark. The logrank p-value is 0.024.

References

1. Firedman N., Geiger D. and Goldszmidt M.:**Bayesian network classifiers.** *Machine Learning* 1997, 29:131-163.
2. Matys V., Fricke E., Geffers R., Gossling E., Haubrock M., Hehl R., Hornischer K., Karas D., Kel AE., Kel-Margoulis OV., Kloos DU., Land S., Lewicki-Potapov B., Michael H., Mnch R., Reuter I., Rotert S., Saxel H., Scheer M., Thiele S., Wingender E.: **TRANSFAC: transcriptional regulation, from patterns to profiles.** *Nucleic Acids Research* 2003, 31(1):374-378.
3. Online Mendelian Inheritance in Man, OMIM. McKusick-Nathans Institute of Genetic Medicine, Johns Hopkins University (Baltimore, MD) and National Center for Biotechnology Information, National Library of Medicine (Bethesda, MD). World Wide Web URL: http://www.ncbi.nlm.nih.gov/omim/.
4. RIKEN Genome Exploration Research Group Phase II Team and the FANTOM Consortium: **Functional annotation of a full-length mouse cDNA collection.** *Nature* 2001, 409(6821):685-690.
5. Benjamini Y. and Hochberg Y.: **Controlling the false discovery rate: a practical and powerful approach to multiple testing.** *Journal of the Royal Statistical Society B* 1995, 57(1):289-300.
6. Storey J. and Tibshirani R.: **Statistical significance for genomewide studies.** *Proceedings of the National Academy of Science USA* 2003, 100(16):9440-9445.
7. Korn E.L., Troendle J.F., McShane L.M. and Simon R.: **Controlling the number of false discoveries: application to high-dimensional genomic data.** *Journal of Statistical Planning and Inference* 2004, 124:379-398.
8. Li S.D., Tagami T., Ho Y.F., Yeang C.H. **Deciphering causal and statistical relations of molecular aberrations and gene expressions in NCI-60 cell lines.** *BMC Systems Biology* 2011, 5:186.
9. The Gene Ontology Database. http://www.geneontology.org/.
10. Joshi-Tope G., Gillespie M., Vastrik I., D'Eustachio P., Schmidt E., de Bono B., Jassal B., Gopinath G.R., Wu G.R., Matthews L., Lewis S., Birney E., Stein L.: **Reactome: a knowledgebase of biological pathways.** *Nucleic Acids Research* 2005, 33:D428-432.
11. BioCarta Database: http://www.biocarta.com/.
12. Schaefer C.F., Anthony K., Krupa S., Buchoff J., Day M., Hannay T., Buetow K.H.: **PID: the Pathway Interaction Database.** *Nucleic Acids Research* 2009, 37:D674-679.
13. Phillips H.S., Kharbanda S., Chen R., Forrest W.F., Soriano R.H., Wu T.D., Misra A., Nigro J.M., Colman H., Soroceanu L., Williams P.M., Modrusan Z., Feuerstein B.G., Aldape K. **Molecular subclasses of high-grade glioma predict**

prognosis, delineate a pattern of disease progression, and resemble stages in neurogenesis., *Cancer Cell* 2006, 9(3):157-173.

14. Ben-Porath I., Thomson M.W., Carey V.J., Ge R., Bell G.W., Regev A., Weinberg R.A. **An embryonic stem cell-like gene expression signature in poorly differentiated aggressive human tumors.** *Nature Genetics* 2008, 40(5):499-507.
15. Cox, D.R.. **Regression models and life-tables.** *Journal of the Royal Statistical Society, Series B* 1972, 34(2):187-220.
16. Verhaak, R.G.W., Hoadley, K.A., Purdom E. et al. **Integrated genomic analysis identifies clinically relevant subtypes of glioblastoma characterized by abnormalities in PDGFRA, IDH1, EGFR, NF1.** *Cancer Cell* 2010, 17:98-110.
17. Noushmehr, H., Weisenberger, D.J., Duefes, K. et al. **Identification of a CpG island methylator phenotype that defines a distinct subgroup of glioma.** *Cancer Cell* 2010, 17:510-522.

Figures

Fig. 1. Consistency of mRNA probe data between two sources (Broad and UNC). The red curve shows the correlation coefficient distribution of intra-gene probes between the two datasets. The blue curve shows the background distribution of correlation coefficients from 10000 random probe pairs of the two datasets.

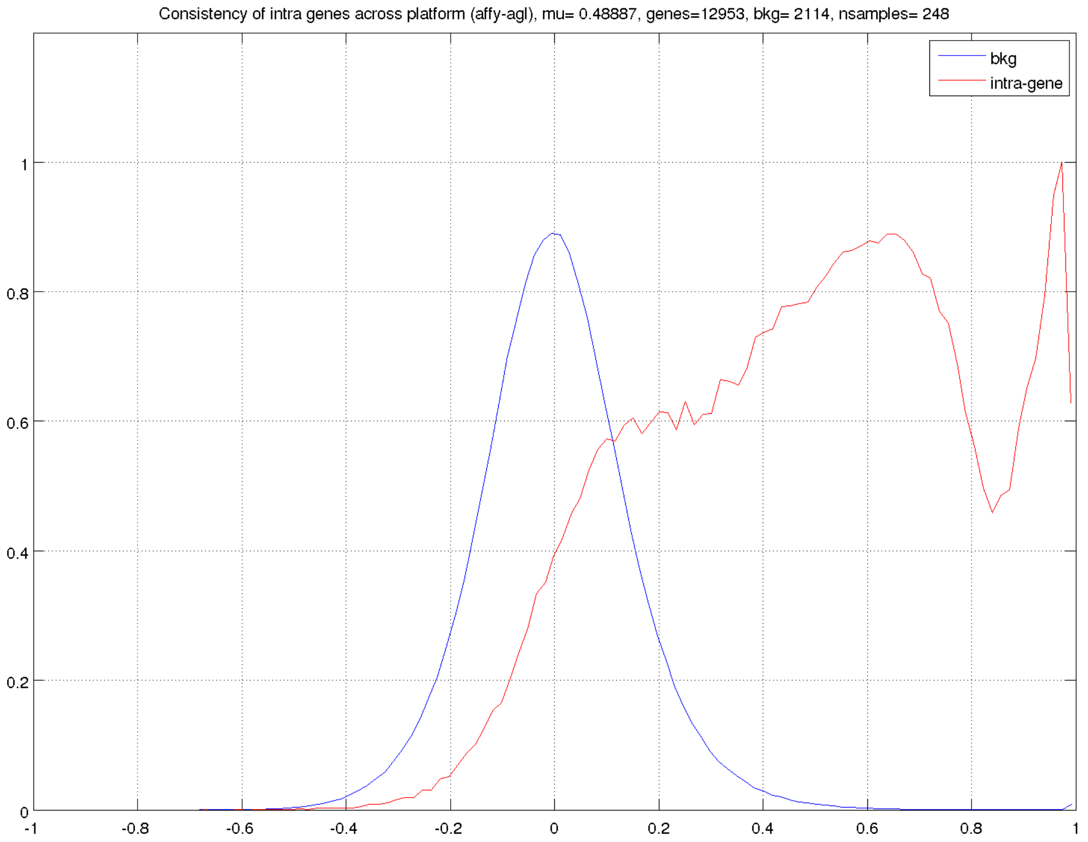

Fig. 2. Consistency of CNV probe data between two sources (Harvard and MSKCC). The red curve shows the correlation coefficient distribution of intra-gene probes between the two datasets. The blue curve shows the background distribution of correlation coefficients from 10000 random probe pairs of the two datasets.

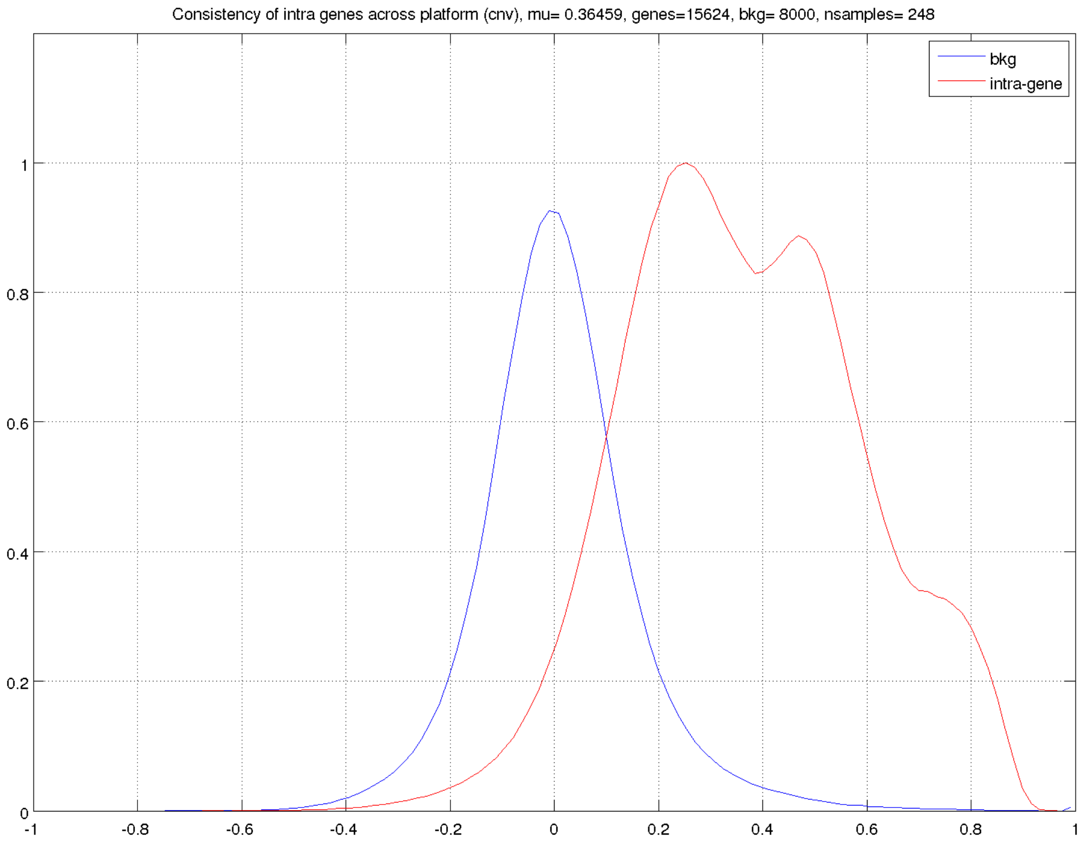

Fig. 3. Auto-correlations of CGH array probes on each chromosome. X axis indicates the base pair distances between probe pairs on the same chromosome. Y axis indicates the mean CNV correlation coefficients of the probe pairs with a fixed distance. Positive and negative directions accommodate probe pairs in the p and q arms respectively. Blue and red curves indicate the data generated by Harvard Medical School (HMS) and Memorial Sloan-Kettering Cancer Center (MSKCC).

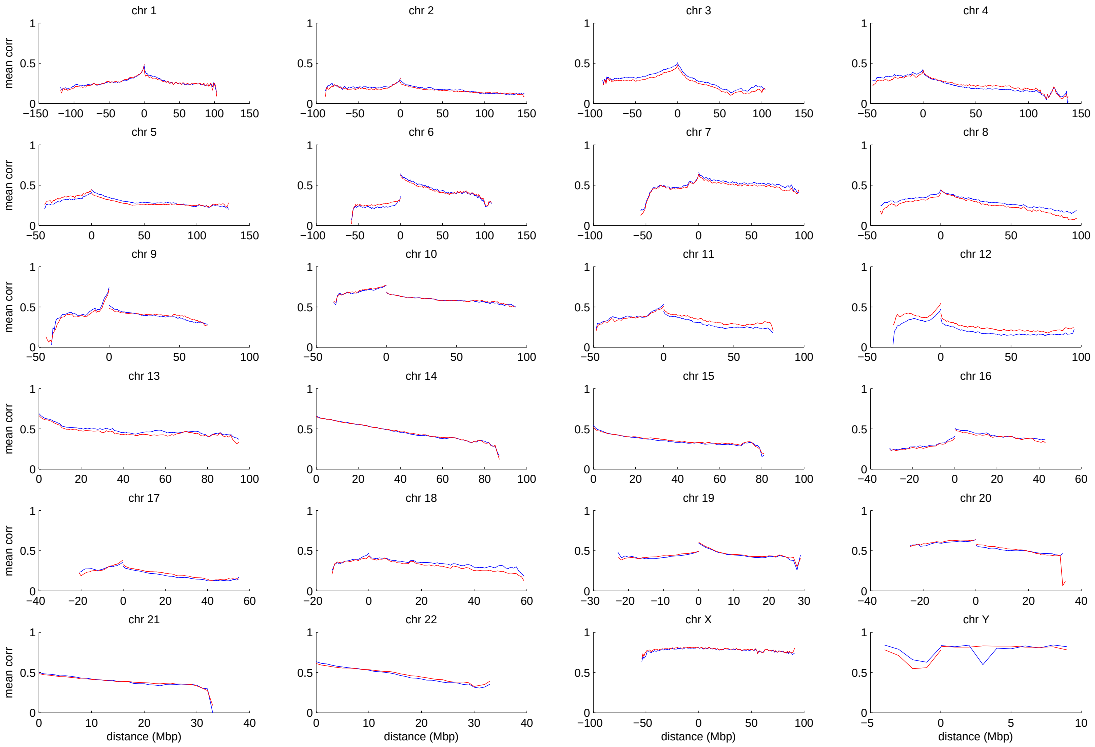

Fig. 4. Cis-acting SNP-mRNA expression pairs. For each panel, the top bar shows the paired alleles of a SNP. Green and red fragments indicate samples carrying two homozygotic alleles respectively, and black fragments indicate samples carrying the heterozygotic allele. The bottom bar shows the normalized expression levels (green: down-regulation, red: up-regulation) of the samples corresponding to the top bar. The identity and location of each SNP and mRNA expression are also displayed.

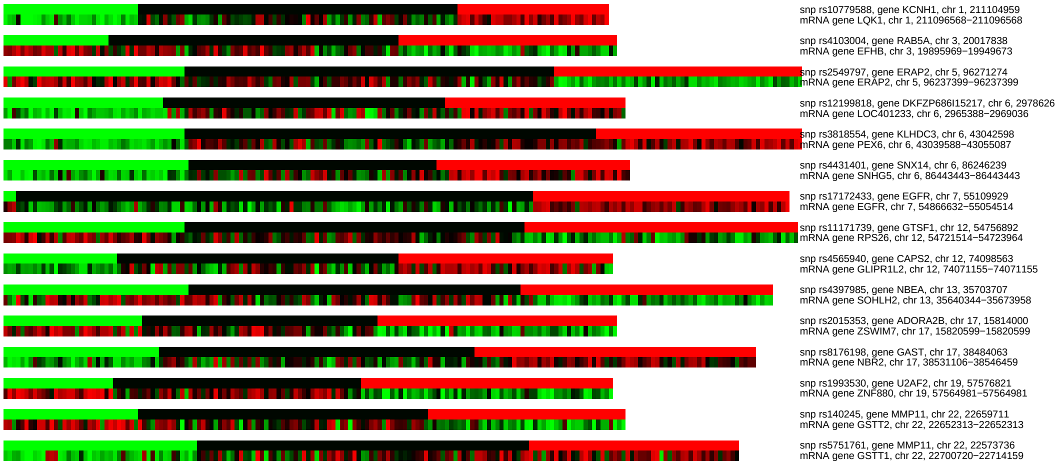

Fig. 5. Comparison of the cis-acting CNV-mRNA associations with the results from six methods of CNV-mRNA data integration. The horizontal axis indicates the rank of genes sorted by scores evaluated from each method. The vertical axis indicates the proportion of genes above a certain rank and present in the CNV cis-acting modules. Red: PCC (Pearson's correlation coefficient), black: SIM (variance test of random effects model), cyan: pSIMCCA (probabilistic canonical correlation analysis), magenta: SPLS (sparse partial least squares regression), blue: SODEGIR (kernal smoothing), and green: GSVD (generalized singular value decomposition).

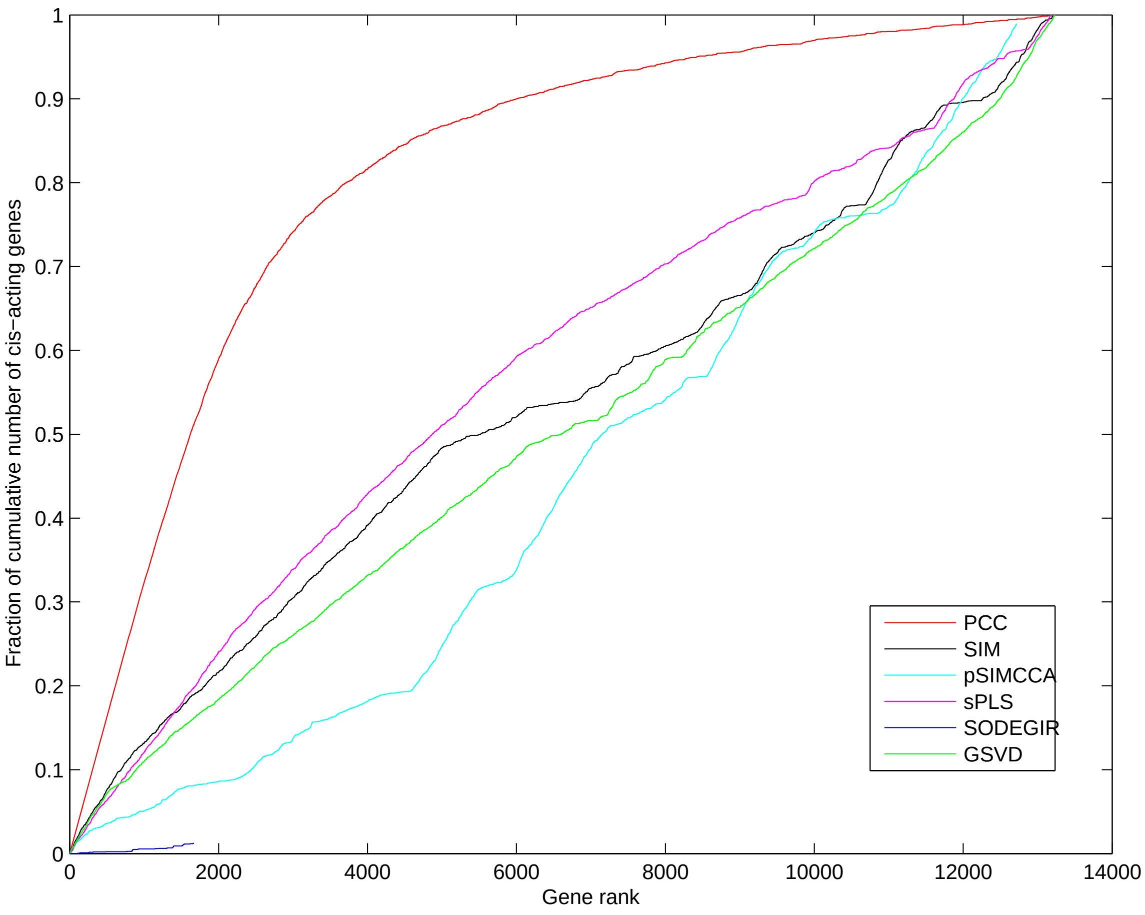

Fig. 6. Cox regression coefficient distributions of 9 association modules (rows) in 5 sample subgroups with distinct tumor types and grades (columns) in the Erasmus data (GSE16011). The five subgroups include OD (oligodendroglial tumors) grade III (44 samples), GBM grade IV (156 samples), A (astrocytoma) grade II (13 samples), A grade III (16 samples), and others (40 samples). Blue curves display the Cox regression coefficient distributions of targets in each association module. Red curves indicate the background distributions of all valid genes in the Erasmus dataset. In each panel, the Kolmogorov-Smirnov p-value of the deviation between the two distributions is reported. A panel is marked with yellow if the corresponding Cox regression coefficient distribution significantly deviates from the background distribution (p-value $\leq 10^{-4}$).

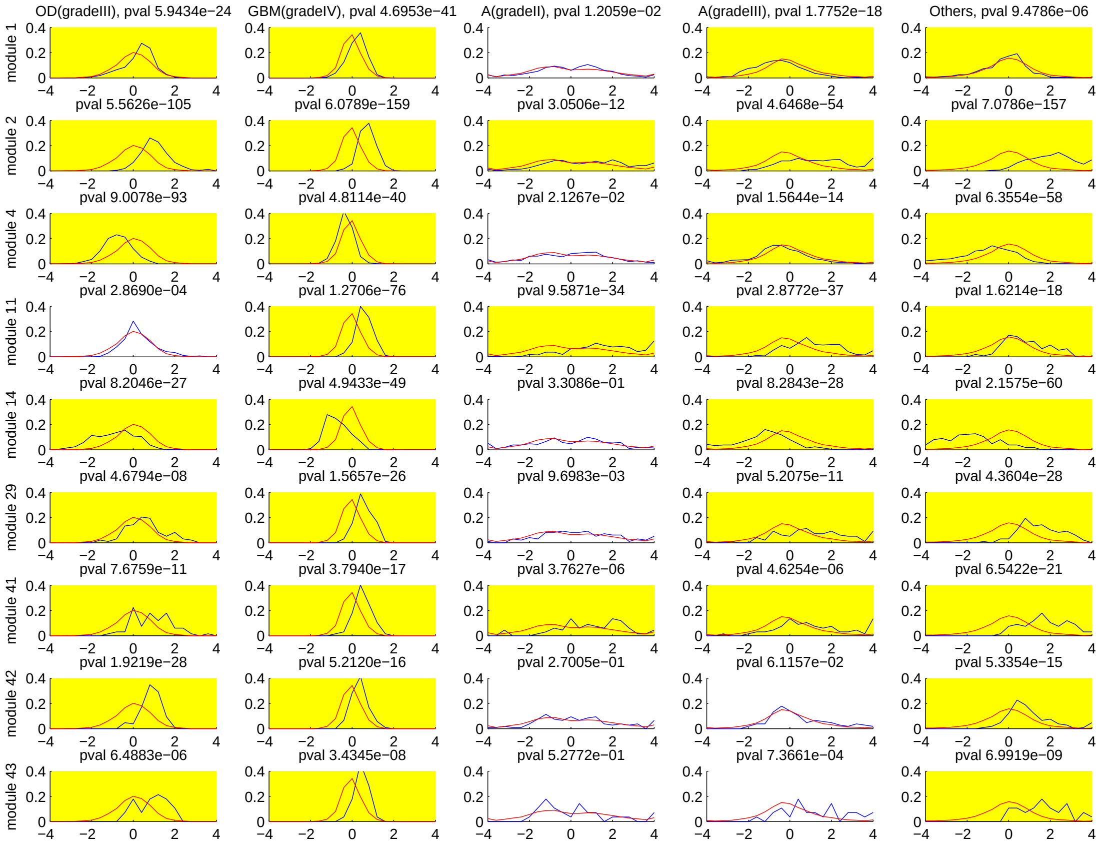

Fig. 7. Kaplan-Meier curves of patients stratified by median target expression values of modules 2 and 14 (columns), in 5 sample subgroups with distinct tumor types and grades (rows) in the Erasmus data (GSE16011). Horizontal axis indicates days and vertical axis indicates the fraction of patients surviving beyond a fixed number of days. Blue curves indicate the survival rates of patients with high expression levels, red curves indicate those of patients with low expression levels.

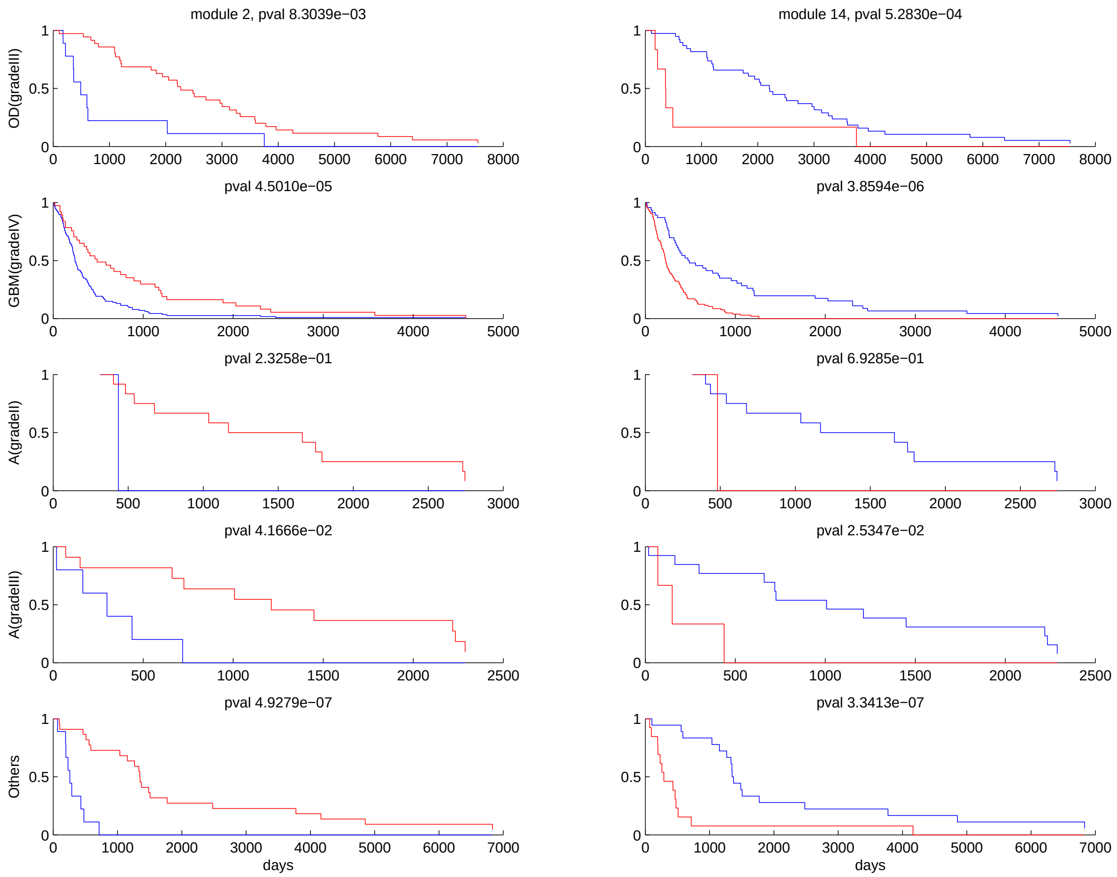

Fig. 8. Cox regression coefficient distributions of 28 association modules in the TCGA GBM data, restricted to G-CIMP positive, G-CIMP negative patients and the entire population. Blue curves display the Cox regression coefficient distributions of targets in each association module. Red curves indicate the background distributions of all valid genes. In each panel, the Kolmogorov-Smirnov p-value of the deviation between the two distributions is reported. A panel is marked with yellow if the corresponding Cox regression coefficient distribution significantly deviates from the background distribution (p-value $\leq 10^{-4}$).

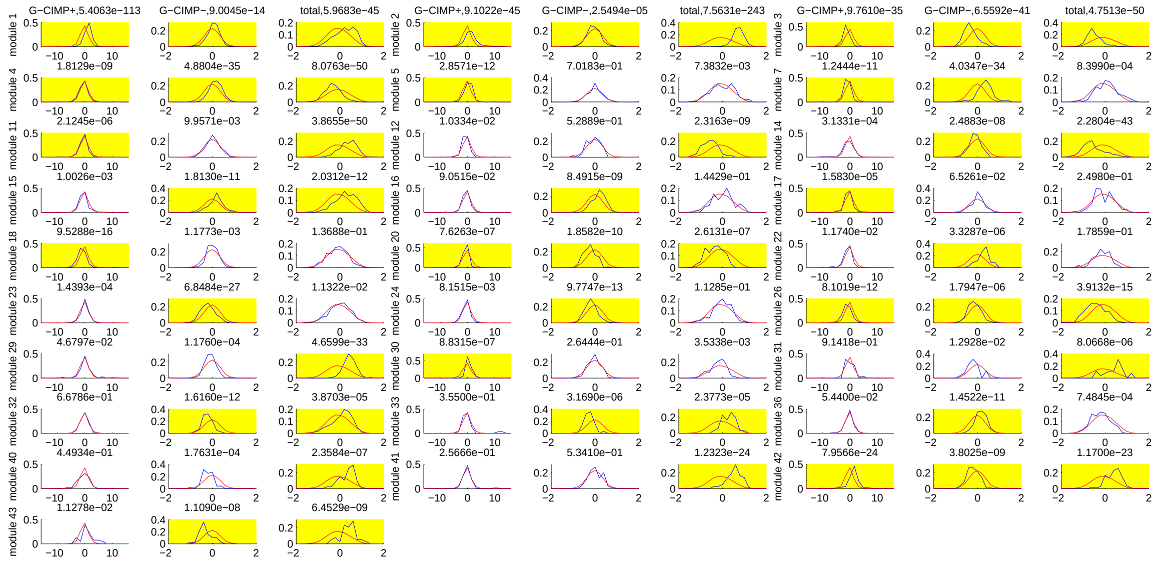

Fig. 9. Kaplan-Meier curves of the TCGA GBM patients stratified by G-CIMP phenotypes and the joint hallmark derived from the median target gene expressions of 16 association modules. Patients are divided into four subgroups: 14 G-CIMP positive tumors with the joint hallmark (black line), 7 G-CIMP positive tumors without the joint hallmark (magenta line), 7 G-CIMP negative tumors with the joint hallmark (blue line), and 217 G-CIMP negative patients without the joint hallmark (red line). $p1$ and $p2$ are the logrank p-values between the subgroups with and without the joint hallmark among the G-CIMP positive and G-CIMP negative patients respectively.

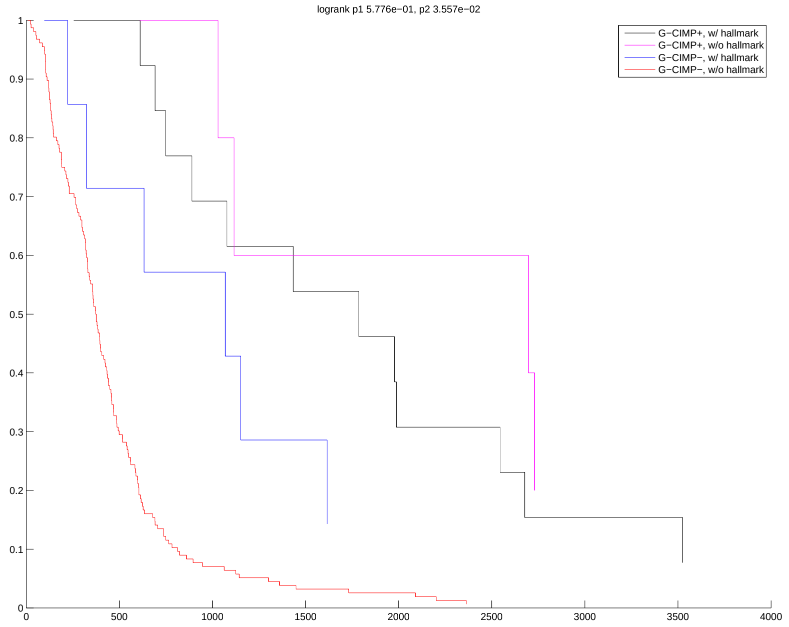

Fig. 10. Kaplan-Meier curves of the TCGA GBM patients stratified by G-CIMP phenotypes and the reduced joint hallmark derived from the median target gene expressions of 4 association modules. Patients are divided into four subgroups: 15 G-CIMP positive tumors with the joint hallmark (black line), 6 G-CIMP positive tumors without the joint hallmark (magenta line), 10 G-CIMP negative tumors with the joint hallmark (blue line), and 214 G-CIMP negative patients without the joint hallmark (red line). $p1$ and $p2$ are the logrank p-values between the subgroups with and without the joint hallmark among the G-CIMP positive and G-CIMP negative patients respectively.

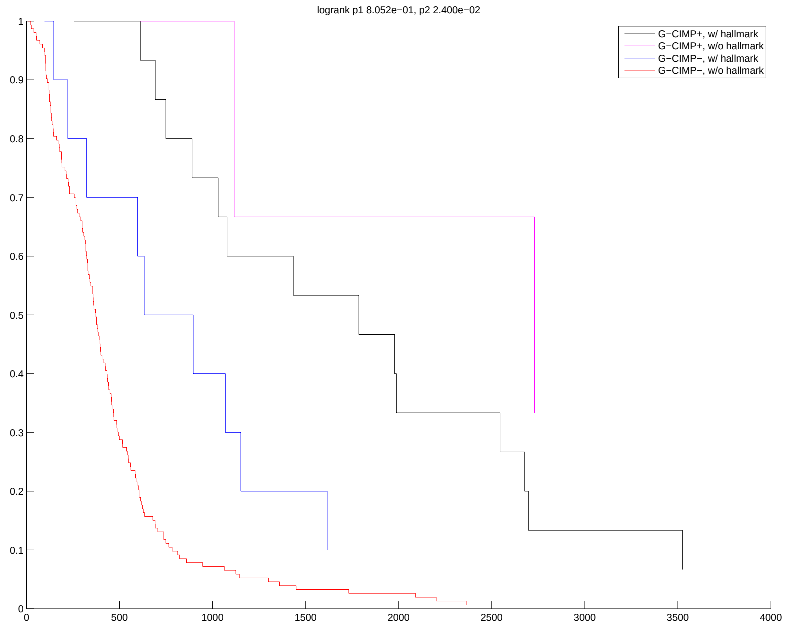

Tables

Table 1. TCGA GBM data included in analysis.

Table 2. Gene symbols, microRNA names and sample IDs in the TCGA GBM data.

Table 3. External datasets of central nervous systems used for validation tests.

Table 4. Threshold values for incorporating associations in the models.

Table 5. The number and fraction of mRNA expressions explained by each type of effectors, and the number and fraction of each type of effectors present in association modules.

Table 6. Prognostic powers of association modules in 4 datasets. Group p-values measure deviations of Cox regression coefficient distributions from the background distributions. Median Cox coefficients and p-values report the Cox regression coefficients and p-values of the median target expression profiles.

Table 7. Cox regression coefficients and p-values of mesenchymal genes in module 2.

Table 8. Overlaps of the four transcriptional subtypes with the binary classes separated by the median target gene expression of each module.

Table 9. Up and down-regulated modules among samples in the G-CIMP positive phenotype and each of the four transcriptional subtypes.

Table 10. Overlaps of the G-CIMP phenotypes with the binary classes separated by the median target gene expression of each module.

Table 11. Coherence of target expression profiles in each module and dataset. The average correlation coefficients and KS test p-values relative to the background distribution of all genes are reported.

Table 12. Associations between effectors and targets in each module and each dataset.

Table 13. Functional enrichment of targets in association modules. N_1: # targets. N_2: # members in the functional class. N_3: # targets belonging to the functional class. p_1: hyper-geometric p-value. p_2: Bonferroni-corrected hyper-geometric p-value.

Table 14. Functional enrichment of association module targets using DAVID and MSigDB. The Bonferroni-corrected p-values for DAVID and false discovery rates for MsigDB are displayed. For each module, we report either the top 20 functional categories/gene sets or the ones whose scores (p-values for DAVID and FDRs for MSigDB) are below 0.01.

Table 15. Enrichment of co-cited effector-target pairs in each module. N_1: # targets. N_2: total # genes co-cited with the effector. N_3: # co-cited targets. p: binomial p-value.

Table 16. Summary information of CNV association modules with microRNA expressions.
